# Pre-surgery gut microbial diversity and abundance are associated with post-surgery onset of cachexia in colorectal cancer patients: the ColoCare Study

**DOI:** 10.1007/s10552-025-02042-y

**Published:** 2025-09-04

**Authors:** Mmadili N. Ilozumba, Maria F. Gomez, Tengda Lin, Caroline Himbert, June L. Round, W. Zac Stephens, Christy A. Warby, Sheetal Hardikar, Christopher I. Li, Jane C. Figueiredo, Victoria Damerell, Gary C. Fillmore, Bartley Pickron, Adetunji T. Toriola, David Shibata, Andreana N. Holowatyj, Christoph Kahlert, Kamya Sankar, Erin M. Siegel, Jolanta Jedrzkiewicz, Biljana Gigic, Doratha A. Byrd, Jennifer Ose, Cornelia M. Ulrich

**Affiliations:** 1 Huntsman Cancer Institute, Salt Lake City, UT USA; 2 Department of Population Health Sciences, University of Utah, Salt Lake City, UT USA; 3 H. Lee Moffitt Cancer Center and Research Institute, Tampa, FL USA; 4 Massachusetts General Hospital, Harvard Medical School, Boston, MA USA; 5 Harvard T.H. Chan School of Public Health, Boston, MA USA; 6 Department of Pathology, University of Utah School of Medicine, Salt Lake City, UT USA; 7 Fred Hutchinson Cancer Center, Seattle, WA USA; 8 Department of Medicine, Cedars-Sinai Medical Center, Samuel Oschin Comprehensive Cancer Institute, Los Angeles, CA USA; 9 Department of General, Visceral, and Transplantation Surgery, Heidelberg University Hospital, Heidelberg, Germany; 10 Washington University School of Medicine in St. Louis, St. Louis, MO USA; 11 Department of Surgery, University of Tennessee Health Science Center, Memphis, TN USA; 12 Vanderbilt University Medical Center, Nashville, TN USA; 13 Department of Information and Communication, Faculty for Media, Information and Design, University of Applied Sciences and Arts, Hannover, Germany

**Keywords:** Gut microbiome, Cachexia, Colorectal cancer

## Abstract

**Background:**

Cachexia accounts for about 20% of all cancer-related deaths and it is indicative of poor prognosis and progressive functional impairment. The role of the gut microbiome in the development of cachexia in colorectal cancer (CRC) patients has not been established.

**Methods:**

Pre-surgical stool samples from *n* = 103 stage I–III CRC patients in the ColoCare Study were analyzed using 16S rRNA gene sequencing (Illumina) to characterize fecal bacteria. We calculated estimates of alpha- and beta-diversity and a priori- and exploratory-selected bacterial relative abundance. Using Fearon criteria, cachexia onset at 6 months post-surgery was defined as > 5% weight loss over the past 6 months and/or body mass index (BMI) of < 20 kg/m^2^ and weight loss of > 2%. Associations of microbial metrics with cachexia onset were estimated using multivariable logistic regression models.

**Results:**

Higher alpha-diversity was positively associated with cachexia onset, with stronger associations in females, patients < 65 years, those receiving adjuvant treatment, consuming high fiber, or with energy intake outside USDA recommendations (*p* < 0.05). *Porphyromonas* (OR = 0.51, 95% CI 0.26–0.89, *p* = 0.03) and *Actinomyces* (OR = 0.72, 95% CI 0.48–1.03, *p* = 0.08) were inversely associated with cachexia, although the association for *Actinomyces* did not reach statistical significance. Stratified analyses revealed a stronger inverse association between *Porphyromonas* and cachexia onset in males, patients with rectal or stage III tumors, those receiving neoadjuvant treatment, physically inactive individuals, and those consuming low fiber. However, these associations did not reach statistical significance (0.05 ≤ *p* < 0.10).

**Conclusion:**

Higher gut microbial alpha-diversity and lower relative abundances of the genera *Porphyromonas* and *Actinomyces* in pre-surgery stool samples were associated with onset of cachexia in CRC patients six months post-surgery. This is the first study to explore a link between the gut microbiome and cachexia in CRC patients, providing novel insights into the biology of cachexia and potential clinical interventions.

**Supplementary Information:**

The online version contains supplementary material available at 10.1007/s10552-025-02042-y.

## Introduction

Cancer cachexia is a complex metabolic syndrome characterized by a continuous loss of skeletal muscle mass which may or may not involve the loss of fat mass and cannot be entirely reversed by nutritional or physical interventions [1]. In general, cachexia accounts for 20% of all cancer-related deaths, and it is indicative of poor prognosis, progressive functional impairment [1–4], and chemotherapy-associated toxicity [5]. Cachexia is highly prevalent in colorectal cancer (CRC), affecting nearly 50% of patients [6]. Aberrant metabolism, a characteristic of cancer cachexia, results from an imbalance between protein synthesis (anabolism) and protein breakdown (hyper-catabolism), leading to systemic inflammation [7–9]. Mechanistically, the development of cachexia is poorly understood, and a more comprehensive insight into the underlying mechanisms of cancer cachexia is crucial. Such knowledge may inform the development of effective treatment strategies and provide a robust foundation for future intervention studies.

**Fig. 1:**
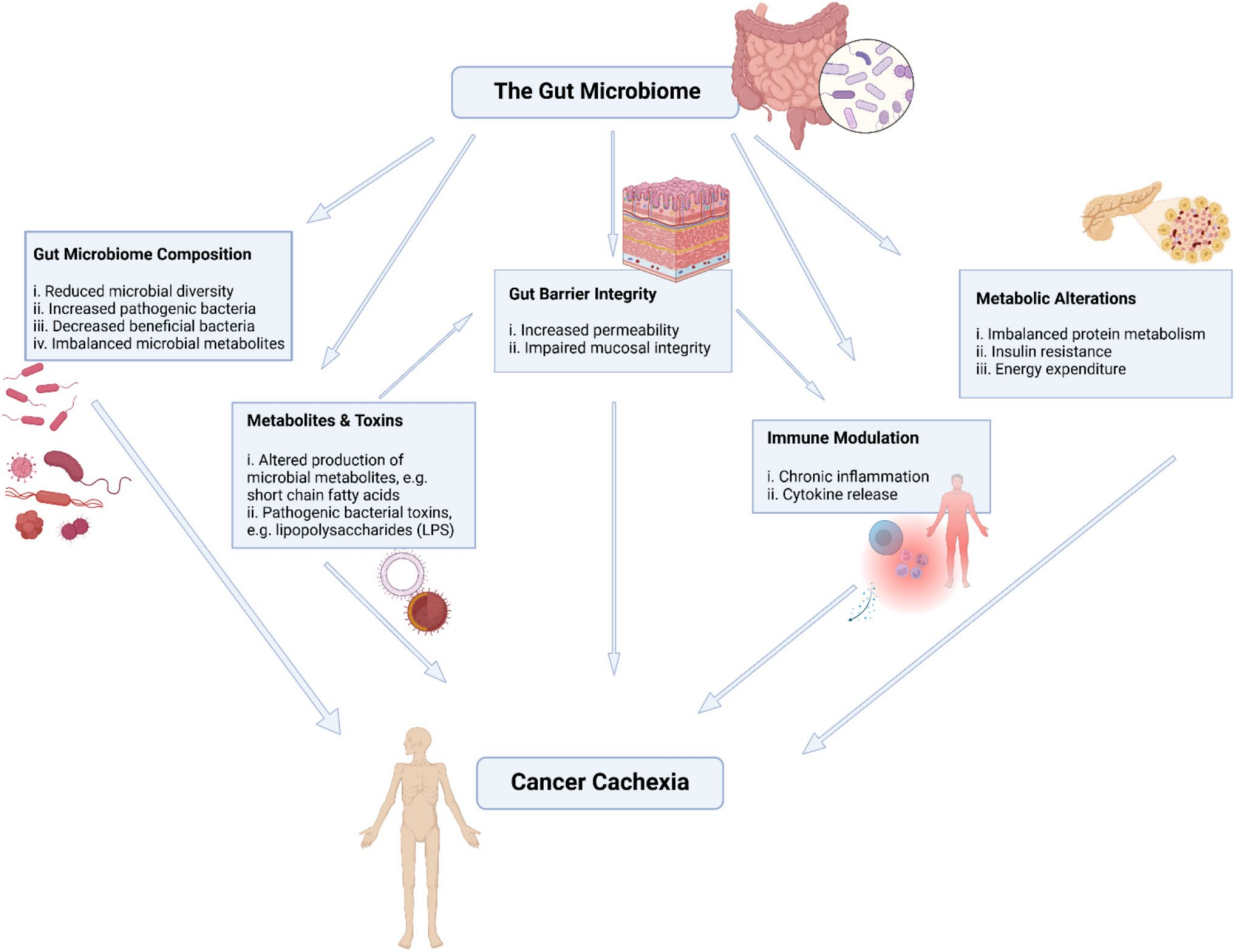
Putative mechanisms linking the gut microbiome to cancer cachexia

The gut microbiome, comprising trillions of microorganisms, has been hypothesized as an underlying mechanism of cancer cachexia as it is implicated in systemic inflammation (Fig. 1) [10]. Pre-clinical evidence regarding associations of the gut microbiome with cancer cachexia mostly includes animal experimental studies, such as those demonstrating that cachectic mice have differential gut microbiota composition and diversity, and increased gut permeability and translocation of pro-inflammatory microbial compounds [10–12]. Compared to control mice, there was a decreased abundance of the genus *Lactobacilli* and an increased abundance of the genus *Parabacteroides* in BaF3 and C26 cachectic tumor-bearing mice [10, 11, 13]. Given that cancer cachexia develops slowly and is a multifactorial syndrome, information gained via animal models may be limited, as these studies involve aggressive, rapidly growing tumors with severe, hard-to-treat cachexia, rather than the early or more manageable stages of cachexia that are more relevant in humans [10].

Human studies investigating the relationship between the gut microbiome and cancer cachexia are limited. In a recent clinical study among a diverse population of *n* = 107 pancreatic, lung, breast, or ovarian cancer patients [14], microbial alpha-diversity did not differ between cachectic and non-cachectic cancer patients or controls, while subtle differences were observed between groups for community structure (beta-diversity). Cachectic cancer patients had higher abundance of *Proteobacteria* and *Veillonella* and lower abundance of *Megamonas* and *Peptococcus* compared to non-cachectic patients[14]. Thus far, no study has examined the association between the gut microbiome and cachexia onset among CRC patients.

Therefore, the goal of this study is to understand the role of the gut microbial alpha- and beta-diversity and abundances of bacteria as a potential biological mechanism underlying the development of cachexia among CRC patients.

## Methods

### Study population

Study participants are from the prospective ColoCare Study (ClinicalTrials.gov NCT02328677), an international cohort of newly diagnosed stage I-IV CRC patients (ICD-10 C18-C20) [15]. The ColoCare Study design has been described elsewhere [15, 16]. The present study comprises data collected from *n* = 103 patients (44 cachectic and 59 non-cachectic) diagnosed with stage I-III CRC enrolled between October 2010 and March 2018 at two study sites at the Heidelberg University Hospital (Heidelberg [HD], Germany) and the Huntsman Cancer Institute ([HCI], Utah, USA) with baseline/pre-surgical available stool samples. Dietary intake was assessed at 6 months post-surgery using self-administered food frequency questionnaire (FFQ). Our study focused on stage I‐III CRC to examine cachexia in early‐stage CRC patients. The study was approved by the institutional review boards of the respective institutions, and all patients provided written informed consent.

### Processing and sequencing of fecal samples

Stool samples were collected by patients at a standardized pre-surgical time point, typically 2 to 3 days prior to bowel preparation for surgery, and suspended directly in RNAlater (Invitrogen), and immediately frozen [17]. In brief, stool samples were self-collected by participants at home using a standardized collection kit, which was consistent across study sites. Each kit contained two stool collection tubes (one “plain” and one RNA later tube, each with corresponding outer and inner tubes), a stool catcher, a stool collection questionnaire, patient instructions, gloves, cold packs, and a labeled black plastic return bag. Participants were instructed to collect the sample using the stool catcher, transfer stool into the tubes, store them in their home freezer immediately, and return them either by mail or in person using the provided frozen cold packs and insulated packaging to maintain sample integrity during transport. The samples were then processed and stored at − 80 °C. Per study protocol, we did not collect any stool samples from patients until at least two weeks after completing neoadjuvant therapy, in order to allow for an appropriate washout period. Most samples were collected prior to the initiation of neoadjuvant treatment. If patients received neoadjuvant treatment, stool samples were collected at least 2 weeks after completion of treatment. Standardized biospecimen collection questionnaires were used for documentation of specific quality control data and covariates, including date and time of stool specimen collection and prior use of antibiotics or NSAIDs [15, 17]. Stool samples were excluded if antibiotic use was reported within 4 weeks of biospecimen collection.

### Microbial DNA/RNA extraction and 16S rRNA sequencing

Total fecal microbial community DNA and RNA were extracted from stool samples using the PowerViral DNA/RNA Kit (Qiagen) according to the manufacturer’s instructions and included 2 minutes of bead-beating at 4 °C with a MiniBeadbeater-16 (BioSpec Products, Bartlesville, OK). Barcoded Illumina 16S rRNA gene sequencing libraries were then prepared by polymerase chain reaction (PCR, in triplicate for each sample) that contained primers with Illumina adapter sequences, barcodes on each primer and sequences targeting the V3 and V4 regions of the 16S rRNA gene. The full oligonucleotide sequences used have been published previously [16]. Triplicate PCRs from each sample were then combined, cleaned up with AxyPrep MAG PCR beads (Axygen), quantified, and evenly multiplexed with other samples as we have previously described [18]. Multiplexed libraries were then sequenced across 2 lanes using an Illumina MiSeq platform at the HCI High-Throughput Genomics and Cancer Bioinformatics Shared Resource. Individual sample libraries that did not have enough reads for analysis (due to random sampling of highly multiplexed libraries) were re-sequenced in a second MiSeq run using the previously prepared sample’s library. Sample controls, mock microbial community DNA, and randomization of samples before extraction, preparation, and sequencing were applied as part of quality control. 2 no template controls (NTC) were run per plate for a total of 4 NTCs. No amplification was detected in the NTCs by agarose gel electrophoresis, but the samples were still cleaned and included in the final multiplexed sequencing library. We recovered very few reads of between 10 and 27 quality reads in each of the 8 NTC samples and no consistent taxonomic assignment among the NTCs, indicating no common background contamination and suggesting these few reads were the result of low-level barcode hopping. For a positive control, we prepared libraries from an 8 species mock community DNA sample (ZymoResearch Cat#D6305) containing even amounts of each species’ DNA. We detected all 8 members with most close to their expected 12% of total, but a slight increase in *Bacillus subtilis* (22%) and decrease in *Pseudomonas aeruginosa reads* (5%) reflecting known low GC-content sequencing bias. All extractions, library preparation, and sequencing were performed at a single laboratory (Round Lab, University of Utah) to prevent variabilities across different laboratories.

### Sequence data processing and taxonomy assignment

Raw sequences were processed to amplicon sequence variants (ASV), and taxonomy was assigned as previously described using the QIIME2 (2020.2) framework [19]. Briefly, paired-end sequences were trimmed of primers, joined, then trimmed to 392 nucleotides, and denoised with deblur. Taxonomy was assigned to representative sequences of each ASV using the Greengenes reference set (13_8) trimmed to the amplified region. ASVs with a prevalence of < 50% and a relative abundance < 0.01% were excluded from exploratory taxonomic analyses. However, a priori-selected bacterial taxa that did not meet these thresholds were retained for targeted hypothesis-driven analyses.

### Cachexia definition and assessment

Cachexia was assessed at 6 months post-surgery. Using Fearon criteria, cachexia was defined as > 5% weight loss over the past 6 months and/or body mass index (BMI) of < 20 kg/m^2^ and weight loss of > 2% [1]. Weight measurements used to calculate percent weight loss for the cachexia definition were obtained from patients’ medical records.

### Statistical analysis

#### Diversity measures and data normalization

Using the Phyloseq package in R, the ASV table was used to calculate alpha-diversity (Shannon index, observed ASVs, and Faith’s Phylogenetic Diversity [PD]) and relative abundance of the bacteria. Alpha- and beta-diversity were calculated based on an empirical rarefaction threshold balancing the number of samples and taxa lost after estimating rarefaction curves. Alpha- and beta-diversity metrics were calculated using rarefied data at a sequencing depth of 11,000 reads per sample. This rarefaction process inherently excludes low-abundance ASVs and minimizes the influence of rare taxa on diversity estimates. Beta-diversity measures were calculated to examine the differences between the samples based on Bray–Curtis, weighted UniFrac, and unweighted UniFrac distance matrices. Alpha- and beta-diversity estimates were calculated based on the average of 10 rarefaction samplings.

#### Genus-level analysis

We selected bacteria at the genus level using an a priori approach based on previous associations with CRC presence and progression, as described in the meta-analysis by Wirbel et al. [20] (which included *Bacteroides, Fusobacterium, Porphyromonas, Parvimonas, Peptostreptococcus, Gemella, Prevotella, Solobacterium, Dialister,* and order Clostridiales) [20, 21] as well as previous associations with cachexia [10, 11, 14, 22–24] (which included *Actinomyces*, *Lactobacillus, Parabacteroides, Bacteroides*, *Veillonella*, *Megamonas*, *Peptococcus, Bifidobacterium*, *Faecalibacterium, Prevotella, Escherichia*). We also conducted exploratory analyses of genera present in 50% of the population at a mean relative abundance of > 0.1% (*n* = 26 taxa). We used the centered log-ratio (CLR) transformation to account for the compositional nature of microbiome data. Relative abundance was modeled continuously. A total of 1,915,421 reads were present, with a mean number of 18,596 reads per sample. Wilcoxon rank sum test was used to compare genus-level relative abundances of a priori-selected bacteria between cachectic and non-cachectic patients.

In a post hoc analysis, we performed a genus-level presence/absence analysis to identify bacterial genera that may be infrequent or low in abundance but differentially detected across groups. Presence was defined as a genus being detected (i.e., non-zero relative abundance) in a given sample. We calculated prevalence as the proportion of samples in which each genus was detected. Genera with a prevalence between 5 to 95% across all samples were included in the analysis.

#### Alpha- and beta-diversity analyses of microbial communities

Alpha-diversity metrics: (1) Shannon index, a measure of the diversity within a community accounting for both the abundance and evenness of species present, (2) Observed Species/ Species Richness, a measure of the number of distinct species present in a sample, and (3) Faith's PD, a measure of the total branch length of a phylogenetic tree encompassing all species present in a sample, incorporating the evolutionary relationships between species were compared between cachectic and non-cachectic patients using ANCOVA and Post hoc Tukey’s Honest Significance Difference test. Standardized alpha-diversity Z-scores were estimated using the formular: *Z* = (*X*−*μ*)/*σ*, where *Z* = Z-score, *X* = observed alpha-diversity value for a specific sample or patient, *μ* = average alpha-diversity value across all samples or the mean of the entire patient distribution, and *σ* = standard deviation of the alpha-diversity values across all samples or the entire patient distribution.

To estimate multivariable differences in beta-diversity by cachectic status, we used a microbiome regression-based kernel associations test (MiRKAT) based on 10,000 permutations. We obtained *p*-values based on kernel similarity matrices for Bray Curtis, unweighted and weighted UniFrac distance, individually and overall. We assessed the Principal Coordinate Analysis (PCoA) plots of bacterial composition differences (Bray Curtis, Unweighted UniFrac, and Weighted UniFrac distances) between cachectic and non-cachectic patients using Permutational ANOVA (PERMANOVA) analysis. In a post hoc analysis, we examined the correlations between beta-diversity (Bray Curtis, Unweighted UniFrac, and Weighted UniFrac distances) with alpha-diversity and relative abundance using PERMANOVA analysis.

#### Multivariable modeling of microbiome and cachexia association

To estimate the associations of the gut microbiome with cachexia onset, we used multivariable logistic models to estimate the odds ratios and 95% confidence intervals. In all models, based on causal diagrams and prior literature, we adjusted for baseline age (< 65 years old, ≥ 65 years old), sex (male or female), stage at diagnosis (I, II, III), tumor site (colon or rectum), smoking status (current, former, never), recruitment center (Heidelberg University Hospital [HD], Germany or Huntsman Cancer Institute [HCI], Salt Lake City, Utah), NSAID/aspirin use (no, yes), C-reactive protein (CRP) levels in mg/L (< 10 mg/L, ≥ 10 mg/L), dietary fiber intake in grams/day (low fiber, high fiber), and energy intake in kcal (Group 1, Group 2). CRP was categorized as (< 10 mg/L, ≥ 10 mg/L) based on clinical cutoff of ≥ 10 mg/L which usually indicates inflammation [25]. Dietary fiber intake was categorized as (low fiber, high fiber) based on USDA recommendation for dietary fiber intake for adults under 50 (25 g of fiber per day for women and 38 g for men) and adults over 50 (21 g for women and 30 g for men) [26]. Energy intake (kcal) was categorized into Group 1 (meets USDA recommendation) and Group 2 (does not meet USDA recommendation) based on USDA guidelines for adults: ages 19–30 (females: 1800–2400 kcal; males: 2400–3000 kcal), ages 31–59 (females: 1600–2200 kcal; males: 2200–3000 kcal), and ages 60 + (females: 1600–2200 kcal; males: 2000–2600 kcal) [27].

We conducted stratified analyses by participant characteristics, including tumor site (colon, rectum), stage (I-II, III), baseline age (< 65 years old, ≥ 65 years old), sex (male, female), adjuvant treatment (no, yes), neoadjuvant treatment (no, yes), physical activity in MET hrs/week (active, ≥ 8.75, inactive, < 8.75), dietary fiber intake in grams/day (low fiber, high fiber), and energy intake in kcal (Group 1, Group 2). We also conducted stratified analysis by recruitment site (HCI, HD). We calculated p-interactions using interaction terms. Sensitivity analyses were conducted excluding patients with antibiotics use (*n* = 19) and patients who received neoadjuvant treatment (*n* = 27), respectively. Statistical significance was defined as *p* < 0.05 and all statistical tests were 2-sided. We accounted for multiple testing using the Bonferroni correction method in the exploratory analyses, with the alpha level set at *p* = 0.0019 (0.05/26). All statistical analyses were conducted using R, version 4.2.2.

## Results

The baseline characteristics of the cohort are described in Table 1. The study population included 44 (43%) cachectic and 59 (57%) non-cachectic CRC patients at 6 months post-surgery. Among cachectic patients, 30 (68%) had rectal cancer and 14 (32%) had colon cancer while among non-cachectic patients, 25 (42%) had rectal cancer while 34 (58%) had colon cancer. At baseline, BMI distributions were similar between cachectic and non-cachectic participants (*p* = 0.09). However, at 6 months, a significantly higher proportion of cachectic vs. non-cachectic participants had a BMI < 25 kg/m^2^ (57% vs. 33%, *p* = 0.001), with a corresponding decline in the proportion with BMI ≥ 30 kg/m^2^ (2% vs. 29%). This shift reflects substantial weight loss over time among those classified as cachectic.

**Table 1:** Study population characteristics, *n* = 103

Characteristics	Cachectic at 6 months post-surgery, *n* (%) 44 (43)	Non-cachectic at 6 months post-surgery, *n* (%) 59 (57)	*p*-value
Age at diagnosis, years old, *n* (%)			0.14
< 65	22 (50)	38 (64)	
≥ 65	22 (50)	21 (36)	
Patient sex, n (%)			0.24
Female	18 (41)	31 (53)	
Male	26 (59)	28 (47)	
Race, *n* (%)			0.38
White	43 (98)	54 (92)	
Asian	0 (0)	1 (2)	
Other	1 (2)	4 (7)	
Ethnicity, n (%)			0.39
Non-Hispanic	44 (100)	58 (98)	
Hispanic	0 (0)	1 (2)	
Study site, *n* (%)			**0.01**
HCI	7 (16)	23 (39)	
HD	37 (84)	36 (61)	
Stage at diagnosis, n (%)			0.44
I	12 (27)	10 (17)	
II	13 (30)	21 (36)	
III	19 (43)	28 (47)	
Tumor site, n (%)			**0.01**
Colon	14 (32)	34 (58)	
Rectum	30 (68)	25 (42)	
Smoking status, *n* (%)			0.08
Non-smoker	19 (44)	26 (44)	
Former smoker	21 (49)	20 (34)	
Current smoker	3 (7)	13 (22)	
BMI at baseline, kg/m^2^, *n* (%)			0.09
< 25	14 (32)	21 (36)	
25–< 30	25 (57)	22 (38)	
≥ 30	5 (11)	15 (26)	
BMI at 6 months, kg/m^2^, n (%)			0.001
< 25	25 (57)	19 (33)	
25–< 30	18 (41)	22 (38)	
≥ 30	1 (2)	17 (29)	
Physical activity, MET hrs/week, *n* (%)			0.58
Active, ≥ 8.75	17 (39)	26 (44)	
Inactive, < 8.75	27 (61)	33 (56)	
Neoadjuvant treatment, n (%)			0.26
No	30 (68)	46 (78)	
Yes	14 (32)	13 (22)	
Adjuvant treatment, *n* (%)			0.27
No	30 (68)	34 (58)	
Yes	14 (32)	25 (42)	
NSAID/Aspirin use, *n* (%)			0.78
No	38 (90)	47 (87)	
Yes	4 (10)	6 (11)	
CRP, mg/L, *n* (%)			0.27
Low CRP, < 10 mg/L	38 (86)	46 (78)	
High CRP, ≥ 10 mg/L	6 (14)	13 (22)	
Dietary fiber intake, grams/day, *n* (%)			0.71
Low fiber	28 (72)	32 (68)	
High fiber	11 (28)	15 (32)	
Energy intake, kcal, *n* (%)			0.82
Group 1	14 (36)	18 (38)	
Group 2	25 (64)	29 (62)	

Missing data: BMI = 1, dietary fiber = 17, energy intake = 17, NSAID/Aspirin use = 8, smoking status = 1

CRP, C-reactive protein (CRP), mg/L; MET—metabolic equivalent per task; HCI, Huntsman Cancer Institute; HD, Heidelberg

CRP was categorized as (< 10 mg/L, ≥ 10 mg/L) based on clinical cutoff of ≥ 10 mg/L which usually indicates inflammation. Dietary fiber intake was categorized as (low fiber, high fiber) based on USDA recommendation for dietary fiber intake for adults under 50 (25 g of fiber per day for women and 38 g for men) and adults over 50 (21 g for women and 30 g for men). Energy intake (kcal) was categorized into Group 1 (meets USDA recommendation) and Group 2 (does not meet USDA recommendation) based on USDA guidelines for adults: ages 19–30 (females: 1800–2400 kcal; males: 2400–3000 kcal), ages 31–59 (females: 1600–2200 kcal; males: 2200–3000 kcal), and ages 60 + (females: 1,600–2200 kcal; males: 2000–2600 kcal)

**Fig. 2:**
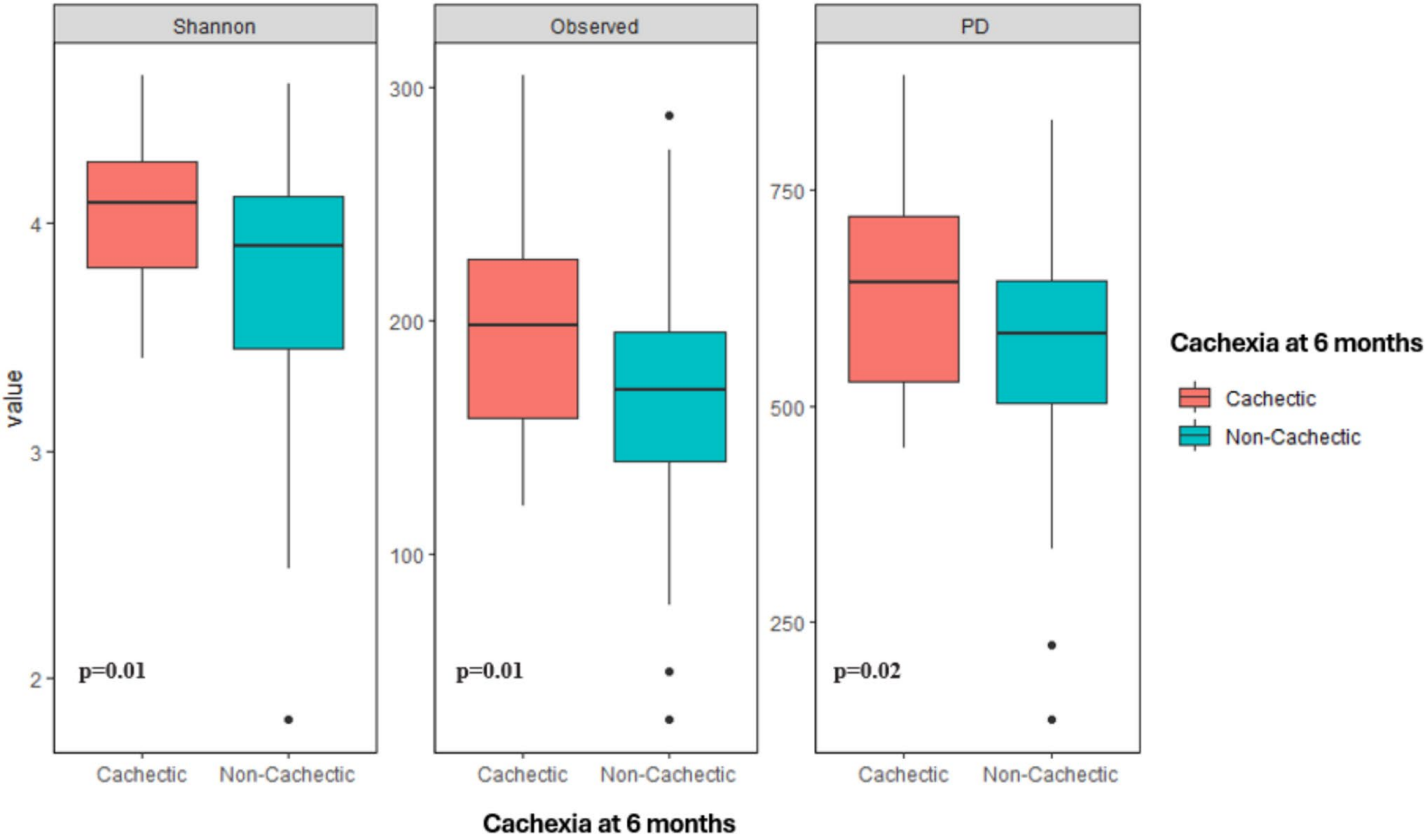
Alpha-diversity between cachectic and non-cachectic patients at 6 months post-surgery. Alpha-diversity metrics: (1) Shannon index, a measure of the diversity within a community accounting for both the abundance and evenness of species present, (2) Observed Species/Species Richness, a measure of the number of distinct species present in a sample, and (3) Faith's PD, a measure of the total branch length of a phylogenetic tree encompassing all species present in a sample, incorporating the evolutionary relationships between species. Alpha-diversity metrics were standardized to a mean of 0 and standard deviation of 1. Standardized alpha-diversity *Z*-scores were estimated: *Z* = (*X*−*μ*)/*σ*, where *Z* = *Z*-score, *X* = observed alpha-diversity value for a specific sample or patient, *μ* = average alpha-diversity value across all samples or the mean of the entire patient distribution, and *σ* = standard deviation of the alpha-diversity values across all samples or the entire patient distribution. Alpha-diversity metrics are presented from left to right in the following order: Shannon index, *p* = 0.01, Observed Species, *p* = 0.01, and Faith’s Phylogenetic Diversity [PD], *p* = 0.02. ANCOVA was used to calculate *p*-values for differences between cachectic and non-cachectic patients adjusting for baseline age (< 65, ≥ 65), sex (male or female), stage at diagnosis (I, II, III), tumor site (colon or rectum), smoking status (current, former, never), recruitment center (Heidelberg University Hospital, Germany or Huntsman Cancer Institute, Salt Lake City), NSAID/Aspirin use (no, yes), C-reactive protein (CRP) levels, mg/L (low CRP, < 10, high CRP, ≥ 10), dietary fiber intake, grams/day (low fiber, high fiber), and energy intake, kcal (Group 1, Group 2). Post hoc Tukey’s HSD test was used to adjust for multiple testing and to test differences between each group. The statistically significant (*P* < 0.05) group comparisons are shown.

Cachectic patients at 6 months post-surgery had higher pre-surgical alpha-diversity compared to non-cachectic patients (Shannon index, *p* = 0.01; Observed species, *p* = 0.01; Faith’s PD, *p* = 0.02), **(**Fig. 2**)**. The logistic regression analyses of associations of alpha-diversity with onset of cachexia overall and stratified by participant characteristics are presented in Table 2. Overall, alpha-diversity was positively associated with cachexia onset (Shannon OR = 2.00, 95% CI = 1.07, 4.19, *p* = 0.04). In stratified analysis, the association between alpha-diversity and cachexia was generally stronger among neoadjuvant treatment naïve patients (Shannon OR = 2.23, *p* = 0.05, *p*-interaction = 0.04); female patients (Observed OR = 4.42, *p* = 0.01, *p*-interaction = 0.02 and Faith’s OR = 4.18, *p* = 0.01, *p*-interaction = 0.02); and patients < 65 years old (Observed OR = 2.93, *p* = 0.02, *p*-interaction = 0.02 and Faith’s OR = 2.58, *p* = 0.03, *p*-interaction = 0.04). Stronger positive associations were also noted among patients who received adjuvant treatment (Shannon OR = 8.12, *p* = 0.03), patients who consumed high dietary fiber (Observed OR = 6.36, *p* = 0.04 and Faith’s OR = 7.13, *p* = 0.03), and patients with energy intake not within USDA recommendations (Shannon OR = 3.10, *p* = 0.02; Observed OR = 2.19, *p* = 0.04; and Faith’s OR = 2.35, *p* = 0.03); however, tests for effect modification (*p*-interaction) were not statistically significant.

**Table 2:** Associations of alpha-diversity^1^ with cachexia onset at 6 months post-surgery overall and stratified by patient characteristics in the ColoCare Study, *n* = 103

		No. cases (%)	OR (95% CI)		*P*_value_^2^	*P*_Strat_^3^	*P*_interaction_
*Shannon*							
Overall			2.00 (1.07, 4.19)		**0.04**		
Tumor site							0.06
	Colon	48 (47)	2.76 (0.96, 7.97)			0.06	
	Rectum	55 (53)	1.68 (0.71, 3.98)			0.24	
Stage							0.25
	Stage I-II	56 (54)	1.60 (0.69, 3.68)			0.25	
	Stage III	47 (46)	2.53 (0.74, 8.69)			0.14	
Sex							0.07
	Male	54 (52)	1.57 (0.58, 4.28)			0.38	
	Female	49 (48)	2.89 (0.96, 8.72)			0.06	
Age, years old							0.12
	< 65	60 (58)	2.51 (0.92, 6.83)			0.07	
	≥ 65	43 (42)	2.44 (0.77, 7.73)			0.13	
Adjuvant treatment							0.25
	No	64 (62)	1.63 (0.73, 3.65)			0.23	
	Yes	39 (38)	8.12 (1.27, 52.07)			**0.03**	
Neoadjuvant treatment							**0.04**
	No	76 (74)	2.23 (1.00, 4.96)			0.05	
	Yes	27 (26)	0.69 (0.11, 4.19)			0.69	
Physical activity, MET hrs/week							0.07
	≥ 8.75	43 (42)	2.84 (0.64, 12.61)			0.17	
	< 8.75	60 (58)	1.73 (0.85, 3.52)			0.13	
Dietary fiber intake, grams/day							0.18
	Low fiber	60 (70)	1.38 (0.56, 3.41)			0.48	
	High fiber	26 (30)	6.25 (0.87, 44.72)			0.07	
Energy intake, kcal							0.68
	Group 1	32 (37)	1.23 (0.24, 6.23)			0.80	
	Group 2	54 (63)	3.10 (1.17, 8.25)			**0.02**	
*Observed*							
Overall			1.63 (0.96, 2.91)		0.08		
Tumor site							0.06
	Colon	48 (47)	2.40 (0.95, 76.07)			0.06	
	Rectum	55 (53)	1.38 (0.67, 2.86)			0.38	
Stage							0.44
	Stage I–II	56 (54)	1.27 (0.59, 2.73)			0.55	
	Stage III	47 (46)	1.61 (0.67, 3.84)			0.29	
Sex							**0.02**
	Male	54 (52)	1.00 (0.47, 2.14)			0.99	
	Female	49 (48)	4.42 (1.40, 14.00)			**0.01**	
Age, years old							**0.02**
	< 65	60 (58)	2.93 (1.17, 7.36)			**0.02**	
	≥ 65	43 (42)	1.12 (0.46, 2.74)			0.81	
Adjuvant treatment							0.50
	No	64 (62)	1.29 (0.63, 2.65)			0.49	
	Yes	39 (38)	2.86 (0.93, 8.79)			0.07	
Neoadjuvant treatment							0.11
	No	76 (74)	1.60 (0.83, 3.08)			0.16	
	Yes	27 (26)	1.12 (0.29, 4.29)			0.87	
Physical activity, MET hrs/week							0.22
	≥ 8.75	43 (42)	1.52 (0.46, 5.00)			0.49	
	< 8.75	60 (58)	1.57 (0.83, 2.98)			0.17	
Dietary fiber intake, grams/day							0.23
	Low fiber	60 (70)	1.20 (0.54, 2.69)			0.65	
	High fiber	26 (30)	6.36 (1.07, 37.67)			**0.04**	
Energy intake, kcal							0.70
	Group 1	32 (37)	1.46 (0.26, 8.14)			0.66	
	Group 2	54 (63)	2.19 (1.04, 4.62)			**0.04**	
*Faith's PD*							
Overall			1.66 (0.97, 3.02)		0.08		
Tumor site							0.05
	Colon	48 (47)	2.56 (0.99, 6.63)			0.05	
	Rectum	55 (53)	1.34 (0.64, 2.77)			0.44	
Stage							0.41
	Stage I-II	56 (54)	1.33 (0.62, 2.86)			0.47	
	Stage III	47 (46)	1.68 (0.67, 4.23)			0.27	
Sex							**0.02**
	Male	54 (52)	1.03 (0.48, 2.20)			0.94	
	Female	49 (48)	4.18 (1.33, 13.17)			**0.01**	
Age, years old							**0.04**
	< 65	60 (58)	2.58 (1.07, 6.21)			**0.03**	
	≥ 65	43 (42)	1.28 (0.51, 3.22)			0.59	
Adjuvant treatment							0.25
	No	64 (62)	1.37 (0.66, 2.85)			0.40	
	Yes	39 (38)	3.18 (0.94, 10.76)			0.06	
Neoadjuvant treatment							0.08
	No	76 (74)	1.73 (0.89, 3.37)			0.11	
	Yes	27 (26)	0.80 (0.18, 3.63)			0.78	
Physical activity, MET hrs/week							0.20
	≥ 8.75	43 (42)	1.62 (0.45, 5.78)			0.46	
	< 8.75	60 (58)	1.59 (0.84, 3.03)			0.16	
Dietary fiber intake, grams/day							0.18
	Low fiber	60 (70)	1.18 (0.52, 3.41)			0.48	
	High fiber	26 (30)	7.13 (1.16, 43.72)			**0.03**	
Energy intake, kcal							0.69
	Group 1	32 (37)	1.41 (0.28, 7.05)			0.68	
	Group 2	54 (63)	2.35 (1.07, 5.15)			**0.03**	

Alpha-diversity metrics: (1) Shannon index, a measure of the diversity within a community accounting for both the abundance and evenness of species present, (2) Observed Species/Species Richness, a measure of the number of distinct species present in a sample, and (3) Faith's PD, a measure of the total branch length of a phylogenetic tree encompassing all species present in a sample, incorporating the evolutionary relationships between species

CRP was categorized as (< 10 mg/L, ≥ 10 mg/L) based on clinical cutoff of ≥ 10 mg/L which usually indicates inflammation. Dietary fiber intake was categorized as (low fiber, high fiber) based on USDA recommendation for dietary fiber intake for adults under 50 (25 g of fiber per day for women and 38 g for men) and adults over 50 (21 g for women and 30 g for men). Energy intake (kcal) was categorized into Group 1 (meets USDA recommendation) and Group 2 (does not meet USDA recommendation) based on USDA guidelines for adults: ages 19–30 (females: 1,800–2,400 kcal; males: 2,400–3,000 kcal), ages 31–59 (females: 1600–2200 kcal; males: 2200–3000 kcal), and ages 60 + (females: 1,600–2,200 kcal; males: 2,000–2,600 kcal)

*Abbreviations*: PD, Faith’s Phylogenetic Diversity; OR, Odds Ratios; CI, Confidence Intervals

^1^Alpha-diversity metrics were standardized to a mean of 0 and standard deviation of 1. Standardized alpha-diversity Z-scores were estimated: *Z* = (*X*−*μ*)/*σ*, where *Z* = *Z*-score, *X* = observed alpha-diversity value for a specific sample or patient, *μ* = average alpha-diversity value across all samples or the mean of the entire patient distribution, and *σ* = standard deviation of the alpha-diversity values across all samples or the entire patient distribution

^2^Adjusted for baseline age, years (< 65, ≥ 65), sex (male or female), stage at diagnosis (I, II, III), tumor site (colon or rectum), smoking status (current, former, never), recruitment center (Heidelberg University Hospital, Germany or Huntsman Cancer Institute, Salt Lake City), NSAID/Aspirin use (no, yes)

^3^Adjusted for baseline age (< 65 years old, ≥ 65 years old), sex (male or female), stage at diagnosis (I-II, III), tumor site (colon or rectum), smoking status (current, former, never), recruitment center (Heidelberg University Hospital, Germany or Huntsman Cancer Institute, Salt Lake City)

**Fig. 3:**
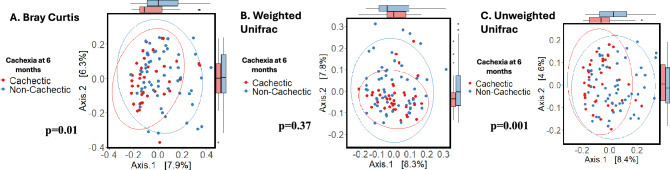
Principal coordinate analysis (PCoA) plots of bacterial composition differences (Bray Curtis, Unweighted UniFrac, and Weighted UniFrac distances) between cachectic and non-cachectic patients, *n* = 103. ^1^Permutational ANOVA (PERMANOVA) analysis was used to determine whether cachectic status differs significantly from each other. ^2^Ellipses indicate 95% confidence intervals. Each dot represents the bacterial composition of cachectic and non-cachectic patients.

**Fig. 4:**
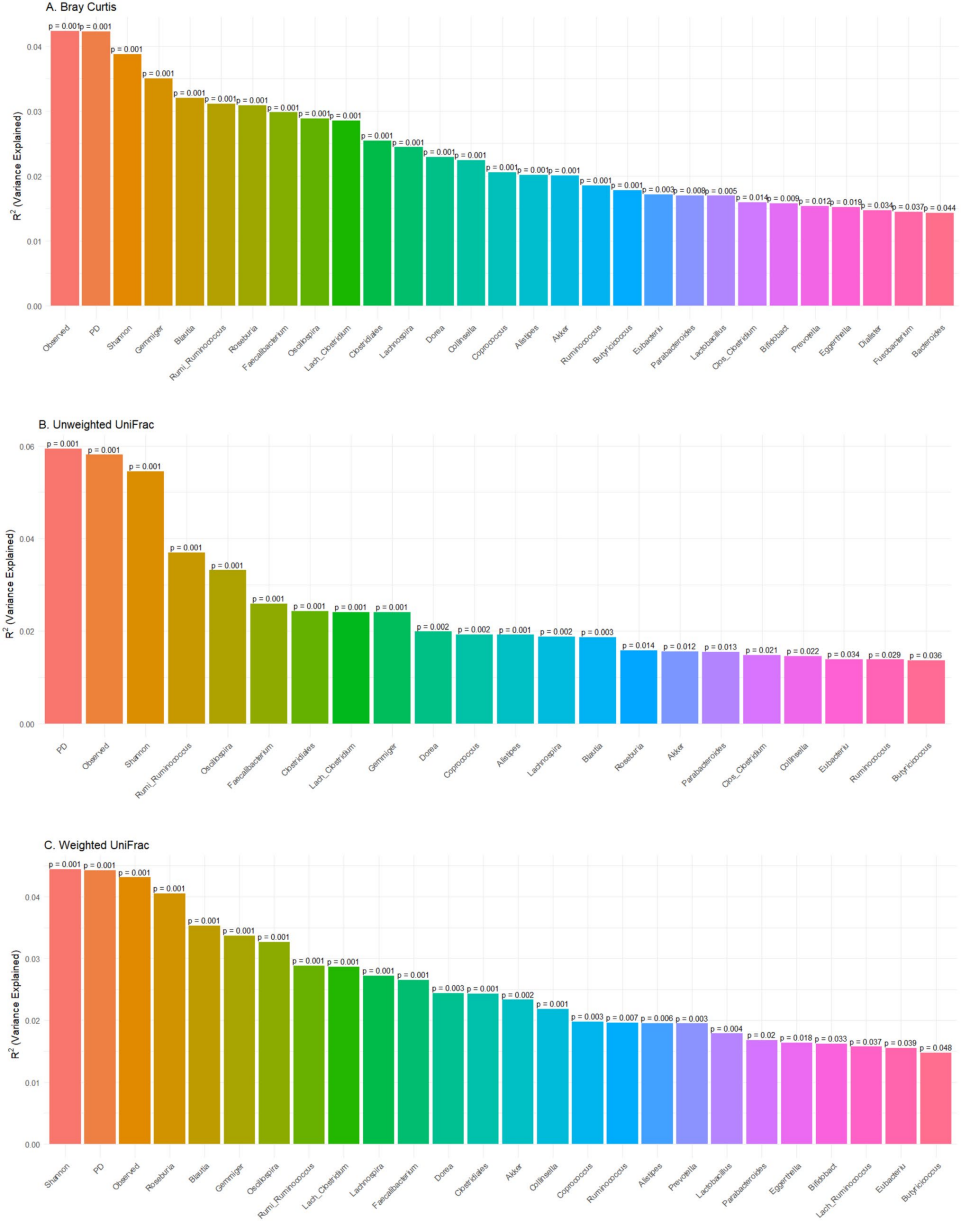
Correlations between beta-diversity (Bray Curtis, Unweighted UniFrac, and Weighted UniFrac distances) with alpha-diversity and relative abundance. ^1^Permutational ANOVA (PERMANOVA) analysis was used adjusting for baseline age (< 65 years old, ≥ 65 years old), sex (male or female), stage at diagnosis (I, II, III), tumor site (colon or rectum), smoking status (current, former, never), recruitment center (Heidelberg University Hospital, Germany or Huntsman Cancer Institute, Salt Lake City), NSAID/ Aspirin use (no, yes), C-reactive protein (CRP) levels, mg/L (low CRP, high CRP,), dietary fiber intake, grams/day (low fiber, high fiber), and energy intake, kcal (Group 1, Group 2). Abbreviations: PD: Faith’s PD; Akker: Akkermansia; Eubacteriu: Eubacterium; Clos_Clostridium: Clostridiaceae (family) Clostridium; Erys_Clostridium: Erysipelotrichaceae (family) Clostridium; Lach_Clostridium: Lachnospiracea (family) Clostridium, Lach_Ruminococcus: Lachnospiracea (family) Ruminococcus; Rumi_Ruminococcus: Ruminococcaceae (family) Ruminococcus

PCoA plots with confidence ellipsoids of bacterial composition differences (Bray Curtis, Unweighted UniFrac, and Weighted UniFrac distances) between cachectic and non-cachectic patients are shown in Fig. 3. There were differences in overall composition and the samples from cachectic and non-cachectic patients clustered separately (Bray Curtis distance, *p* = 0.01 and unweighted Unifrac distance, *p* = 0.001). In multivariable analysis, there were differences in overall microbiome composition between cachectic and non-cachectic patients. The first principal coordinate axes of Bray Curtis distance was inversely associated with cachexia onset. The first axes of unweighted Unifrac distance was positively associated with cachexia onset (Table 3). The correlations between beta-diversity (Bray Curtis, Unweighted UniFrac, and Weighted UniFrac distances) with alpha-diversity and relative abundance are shown in Fig. 4.

**Table 3:** Associations of the first three principal coordinate axes of Bray Curtis, Unweighted UniFrac, and Weighted UniFrac distances with onset of cachexia, *n* = 103

Beta-diversity axes	OR (95% CI)^1^	*P*-value
BC PC1	0.49 (0.24, 0.91)	**0.03**
BC PC2	0.88 (0.51, 1.51)	0.65
BC PC3	0.92 (0.57, 1.49)	0.73
Unweighted PC1	2.43 (1.26, 5.14)	**0.01**
Unweighted PC2	1.15 (0.70, 1.91)	0.58
Unweighted PC3	1.37 (0.79, 2.37)	0.25
Weighted PC1	0.83 (0.48, 1.42)	0.50
Weighted PC2	0.66 (0.33, 1.20)	0.19
Weighted PC3	0.93 (0.56, 1.51)	0.76

^1^Principal coordinates were standardized to a mean of 0 and standard deviation of 1. Adjusted for baseline age (< 65 years old, ≥ 65 years old), sex (male or female), stage at diagnosis (I, II, III), tumor site (colon or rectum), smoking status (current, former, never), recruitment center (Heidelberg University Hospital, Germany or Huntsman Cancer Institute, Salt Lake City), NSAID/ Aspirin use (no, yes), C-reactive protein (CRP) levels, mg/L (low CRP, high CRP,), dietary fiber intake, grams/day (low fiber, high fiber), and energy intake, kcal (Group 1, Group 2)

BC: Bray Curtis, PC: Principal coordinate axis, OR: Odds ratios, CI: Confidence intervals

In multivariable a priori analyses of selected bacteria previously associated with CRC and cachexia from the literature (Table 4) [10, 11, 14, 20–24], considered per 1-unit increase in the centered log-ratio (CLR) transformed abundance, *Porphyromonas* and *Actinomyces* were inversely associated with cachexia onset, although the association for *Actinomyces* did not reach statistical significance (OR = 0.51, 95% CI = 0.26, 0.89; *p* = 0.03 and OR = 0.72, 95% CI = 0.48, 1.03; *p* = 0.08, respectively). In multivariable *exploratory* analyses of genera present in 50% of the bacterial population at a mean relative abundance of > 0.1% (Table 5), considered per 1-unit increase in the CLR transformed abundance, *Erysipelotrichaceae (family) Clostridium* showed a positive association with cachexia onset while *Turicibacter* showed a negative association with cachexia onset, although these associations were not statistically significant (0.05 ≤ *p* < 0.10).

**Table 4:** Associations^1^ of a priori-selected bacterial relative abundances^2^ with cachexia onset at 6 months post-surgery in the ColoCare Study, *n* = 103

	Mean (min–max)	OR (95% CI)	*P*-values
*A priori-selected bacteria associated with CRC*			
*Bacteroides*	9.30 (0.00, 64.49)	0.78 (0.50, 1.19)	0.26
Clostridiales (Order)	73.16 (18.47, 96.49)	1.17 (0.66, 2.11)	0.59
*Dialister*	0.88 (0.00, 6.39)	0.88 (0.59, 1.31)	0.52
*Fusobacterium*	0.18 (0.00, 10.77)	0.98 (0.66, 1.44)	0.93
*Parvimonas*	0.14 (0.00, 6.68)	0.95 (0.58, 1.57)	0.85
*Peptostreptococcus*	0.35 (0.00, 17.43)	1.08 (0.72, 1.64)	0.70
*Porphyromonas*	0.10 (0.00, 3.36)	0.51 (0.26, 0.89)	**0.03**
*Prevotella*	2.81 (0.00, 48.70)	1.00 (0.72, 1.64)	0.87
*A priori-selected bacteria associated with cachexia in non-CRC patients*			
*Actinomyces*	0.05 (0.00, 0.88)	0.72 (0.48, 1.03)	0.08
*Lactobacillus*	0.70 (0.00, 36.87)	1.00 (0.70, 1.44)	0.98
*Parabacteroides*	0.89 (0.00, 4.83)	0.93 (0.55, 1.58)	0.79
*Bacteroides*	9.30 (0.00, 64.49)	0.78 (0.50, 1.19)	0.26
*Veillonella*	0.18 (0.00, 5.66)	0.81 (0.53, 1.23)	0.32
*Megamonas*	0.17 (0.00, 22.30)	0.68 (0.04, 2.00)	0.62
*Peptococcus*	0.06 (0.00, 1.73)	0.80 (0.30, 2.37)	0.66
*Bifidobacterium*	4.12 (0.00, 27.43)	1.31 (0.96, 1.85)	0.10
*Faecalibacterium*	8.89 (0.00, 31.58)	1.24 (0.88, 1.81)	0.23
*Prevotella*	2.81 (0.00, 48.70)	1.00 (0.72, 1.38)	0.99
*Escherichia*	0.06 (0.00, 4.86)	1.46 (0.74, 4.16)	0.36

^1^Odds ratios and 95% confidence intervals were estimated using multivariable logistic regression adjusted for baseline age (< 65 years old, ≥ 65 years old), sex (male or female), stage at diagnosis (I, II, III), tumor site (colon or rectum), smoking status (current, former, never), recruitment center (Heidelberg University Hospital, Germany or Huntsman Cancer Institute, Salt Lake City), NSAID/ Aspirin use (no, yes), C-reactive protein (CRP) levels, mg/L (low CRP, high CRP), dietary fiber intake, grams/day (low fiber, high fiber), and energy intake, kcal (Group 1, Group 2)

^2^Bacteria relative abundances were transformed with the centered log-ratio transformation method. Includes all genera identified. Bacteria present in ≥ 50% of samples at an average relative abundance of ≥ 0.01%

PD, Faith’s Phylogenetic Diversity; OR, Odds Ratios; CI, Confidence Intervals

**Table 5:** Associations^1^ of exploratory bacterial relative abundances^2^ with cachexia onset at 6 months post-surgery in the ColoCare Study, *n* = 103

	Mean (min–max)	OR (95% CI)	*P*-values^3^
*Exploratory-selected bacteria*			
*Akkermansia*	1.63 (0.00, 28.03)	0.83 (0.59, 1.14)	0.26
*Alistipes*	1.11 (0.00, 11.54)	1.04 (0.73, 1.46)	0.84
*Anaerostipes*	0.32 (0.00, 5.53)	1.27 (0.82, 2.04)	0.29
*Biloxi*	0.17 (0.00, 1.34)	0.95 (0.54, 1.67)	0.86
*Blautia*	14.51 (1.17, 42.81)	0.92 (0.53, 1.58)	0.76
*Butyricicoccus*	0.30 (0.00, 1.61)	1.23 (0.71, 2.20)	0.46
*Clostridiaceae (family) Clostridium*	1.84 (0.00, 37.33)	1.18 (0.80, 1.78)	0.40
*Collinsella*	2.57 (0.00, 22.83)	1.12 (0.81, 1.59)	0.49
*Coprococcus*	5.45 (0.28, 19.75)	1.06 (0.62, 1.82)	0.83
*Dorea*	3.54 (0.00, 14.70)	1.15 (0.71, 2.02)	0.59
*Eggerthella*	0.12 (0.00, 1.49)	1.36 (0.90, 2.14)	0.15
*Erysipelotrichaceae (family) Clostridium*	0.11 (0.00, 4.01)	1.62 (1.00, 2.76)	0.06
*Eubacterium*	0.56 (0.00, 4.80)	1.05 (0.76, 1.46)	0.76
*Faecalibacterium*	8.89 (0.00, 31.58)	1.24 (0.88, 1.81)	0.23
*Gemmiger*	4.72 (0.00, 24.84)	1.12 (0.81, 1.57)	0.50
*Lachnospiracea (family) Clostridium*	0.61 (0.00, 10.67)	0.82 (0.54, 1.20)	0.31
*Lachnospiracea (family) Ruminococcus*	0.46 (0.00, 3.04)	0.78 (0.50, 1.18)	0.24
*Lachnospira*	1.49 (0.00, 10.07)	1.15 (0.76, 1.77)	0.51
*Oscillospira*	4.33 (0.00, 33.15)	1.03 (0.68, 1.60)	0.88
*Phascolarctobacterium*	0.55 (0.00, 4.62)	0.99 (0.54, 1.74)	0.96
*Roseburia*	8.03 (0.00, 29.75)	1.14 (0.83, 1.60)	0.42
*Ruminococcaceae (family) Ruminococcus*	8.77 (0.00, 25.88)	1.32 (0.95, 1.89)	0.11
*Ruminococcus*	3.72 (0.00, 52.56)	0.86 (0.52, 1.42)	0.57
*Streptococcus*	1.19 (0.00, 25.10)	0.99 (0.69, 1.43)	0.96
*Sutterella*	0.59 (0.00, 3.20)	0.98 (0.61, 1.58)	0.95
*Turicibacter*	0.23 (0.00, 6.11)	0.61 (0.34, 1.04)	0.08

^1^Odds ratios and 95% confidence intervals were estimated using multivariable logistic regression adjusted for baseline age (< 65 years old, ≥ 65 years old), sex (male or female), stage at diagnosis (I, II, III), tumor site (colon or rectum), smoking status (current, former, never), recruitment center (Heidelberg University Hospital, Germany or Huntsman Cancer Institute, Salt Lake City), NSAID/ Aspirin use (no, yes), C-reactive protein (CRP) levels, mg/L (low CRP, high CRP), dietary fiber intake, grams/day (low fiber, high fiber), and energy intake, kcal (Group 1, Group 2)

^2^Bacteria relative abundances were transformed with the centered log-ratio transformation method. Includes all genera identified. Bacteria present in ≥ 50% of samples at an average relative abundance of ≥ 0.01%

^3^*P*-values are corrected for multiple testing using the Bonferroni correction method at a significance level of 0.0019 (0.05/ 26)

OR: Odds Ratios, CI: Confidence intervals

Supplemental Table S1 shows the associations of a priori-selected bacterial relative abundances with cachexia onset at 6 months stratified by patient characteristics (*p* < 0.05 and 0.05 ≤ *p* < 0.10). For *Porphyromonas*, stronger inverse associations were observed in males and patients with neoadjuvant treatment, rectal tumors, stage III tumors, inactive patients, and patients who consumed low dietary fiber. For *Actinomyces*, stronger inverse association was found in patients < 65 years old. For *Bifidobacterium*, stronger positive association was found among females and inactive patients. For *Veillonella*, stronger inverse association was found among patients ≥ 65 years old, patients who did not receive adjuvant treatment and patients with energy intake within USDA recommendations. For *Bacteroides,* stronger inverse associations were observed in females. For *Faecalibacterium*, stronger positive associations were found in inactive patients.

In the stratified analysis by recruitment site, stronger inverse associations were observed for *Porphyromonas,* and *Veillonella* in HD (OR = 0.46; *p* = 0.04, and OR = 0.63; *p* = 0.09, respectively) and for *Parabacteroides* in HCI (OR = 0.15; *p* = 0.07) (Supplemental Table S3). Sensitivity analyses excluding patients with antibiotic use (Supplemental Tables S4–S6) and patients who received neoadjuvant treatment (Supplemental Tables S7–S9) did not affect study findings. In the genus level presence/absence analysis with genera prevalence between 5 and 95% across all samples, *Fusobacterium*, *Eubacterium,* and *Clostridium* were inversely associated with cachexia onset while *Lactococcus*, and *Akkermansia* were positively associated with cachexia onset (Supplemental Table S10). Comparison of genus-level relative abundances of a priori-selected bacteria between cachectic and non-cachectic patients are shown in Supplemental Figure S1.

## Discussion

We observed that pre-surgical alpha-diversity was positively associated with cachexia onset. Additionally, we observed stronger associations of alpha-diversity with cachexia among several clinicopathological and demographic characteristics, including neoadjuvant treatment-naïve patients, female patients, and patients under 65 years old. Furthermore, in our analyses of bacterial taxa, we identified that the abundances of multiple bacteria at the genus level were associated with cachexia onset, including some bacteria that were previously established to be associated with CRC and cachexia, including *Porphyromonas* and *Actinomyces* [20, 21, 24]. Stronger associations of bacterial relative abundances with cachexia were observed among several patients’ clinicopathological and demographic characteristics. For *Porphyromonas*, stronger inverse associations were observed in male patients, physically inactive patients, those who received neoadjuvant treatment, patients with rectal tumors, stage III tumors, and those with low dietary fiber intake. For *Actinomyces*, stronger inverse association was found in patients < 65 years old.

In the present study, we observed that cachectic patients with CRC at six months post-surgery had higher pre-surgical alpha-diversity compared to non-cachectic patients with alpha-diversity being positively associated with cachexia onset, which was contrary to our study hypothesis. The exact reason for the presence of higher alpha-diversity in cachectic patients and reversed direction in association is not entirely clear but may reflect several underlying factors. One possibility is that the higher baseline alpha-diversity in cachectic patients could represent a distinct microbial environment with enrichment of pro-inflammatory taxa, contributing to metabolic dysregulation or immune modulation, potentially influencing cachexia onset. Additionally, the cachectic group had a higher proportion of overweight individuals and a lower proportion of individuals with obesity compared to the non-cachectic group. Emerging evidence suggests that individuals with overweight, compared to those with obesity, may harbor a more diverse gut microbiota. For example, studies have shown that gut microbial alpha-diversity is typically reduced in individuals with obesity and is associated with greater adiposity, dyslipidemia, impaired glucose homeostasis, and systemic low-grade inflammation [28–30]. Our findings suggest that baseline adiposity may shape the gut microbiome’s influence on cachexia risk, warranting further research into how it modulates microbiota features and their contribution to cachexia development. Moreover, skeletal muscle mass and visceral adipose tissue, which are key factors in cachexia development may have played a role, although we lacked direct measures of body composition. These factors could have masked or modified the expected relationship between alpha-diversity and cachexia onset, warranting further investigation in future studies with comprehensive body composition data. Cachectic patients in the study had a higher percentage of rectal tumors compared to non-cachectic patients, suggesting that rectal tumors harbor distinct microbial signatures due to their proximity to the anal microbiome potentially enriching diversity in these patients. Although systemic inflammation is a hallmark of cancer cachexia, 86% of cachectic patients in our cohort had CRP < 10 mg/L, indicating limited acute inflammation. This suggests that cachexia may be driven by chronic low-grade inflammation not reflected by standard CRP thresholds, underscoring the need for broader biomarker panels in future studies. Parenteral and/or enteral nutrition and intake of dietary supplements such as probiotics may alter alpha-diversity and microbiome abundance [31–34]. However, it is unknown whether cachectic patients in our cohort received parenteral and/or enteral nutrition and dietary supplements. Further research is needed to better understand these findings and the underlying biological mechanisms driving the relationship between microbiome diversity and cachexia in CRC.

We observed that a priori-selected bacteria *Porphyromonas* was inversely associated with cachexia onset. *Porphyromonas* is a genus of gram-negative, anaerobic bacteria commonly found in the oral cavity and gastrointestinal tract [35, 36]. While some *Porphyromonas* species, like *Porphyromonas gingivalis*, are known for their pro-inflammatory roles and have been associated with decreased cancer-specific survival in CRC patients [35–38], other species might exhibit different behaviors depending on the environment. The inverse association of *Porphyromonas* with cachexia onset in our study could be explained by several potential mechanisms, reflecting complex host-microbe interactions. Certain *Porphyromonas* species might modulate the immune response in a way that reduces systemic inflammation, potentially counteracting processes that lead to muscle wasting in cachexia. Some *Porphyromonas* species may contribute to the production of microbiota-derived metabolites such as short-chain fatty acid (SCFA) like butyrate produced during the microbial fermentation of dietary fibers which influences gut health in addition to their anti-inflammatory and immune-modulatory properties [39, 40]. *Porphyromonas* abundance may reflect a compensatory microbial shift aimed at counterbalancing inflammatory processes or other microbial imbalances, particularly in males, physically inactive patients, or those with rectal tumors and stage III tumors. Further studies are needed to unravel species-specific roles and functional implications of *Porphyromonas* in CRC-related cachexia.

Longitudinal epidemiological studies on the associations of the gut microbiome with cancer cachexia in CRC patients are currently lacking. In a recent cross‐sectional case–control study, comprising of 107 pancreatic, lung, breast, or ovarian cancer patients [14], no differences in microbial alpha-diversity were detected between cachectic and non-cachectic cancer patients or controls. Differences in cancer type and study design may account for the discrepancies in our findings regarding alpha-diversity. Cachectic cancer patients had more abundance of *Veillonella* compared to non-cachectic patients [14]. However, we observed no differences in the relative abundance of *Veillonella* between cachectic and non-cachectic patients. Additionally, we found stronger inverse associations between *Veillonella* and cachexia onset among patients ≥ 65 years old, patients who did not receive adjuvant treatment and patients with energy intake within USDA recommendations. *Veillonella* species are generally considered to have a neutral to potentially beneficial role in the gut microbiome. They are known to contribute to the fermentation of lactate into SCFAs like acetate and propionate, which have anti-inflammatory properties and help maintain gut health [41, 42]. *Veillonella* might be involved in metabolizing lactate produced during exercise, which could potentially influence muscle metabolism and recovery [43]. Studies have suggested that *Veillonella* and *Actinomyces* may help balance the microbial ecosystem by interacting with other bacteria to produce metabolites that help maintain metabolic balance thereby supporting an anti-inflammatory environment [41, 42, 44]. The precise roles of *Veillonella* and *Actinomyces* in CRC cachexia remain an area for further investigation.

Our study had several strengths. To date, this is the first study leveraging pre-surgical fecal samples to investigate the associations of the gut microbiome with cachexia onset six months post-surgery in CRC patients. Fecal sampling is non-invasive and provides a representation of the microbial communities residing within the gastrointestinal tract. One of the major strengths of our study is its prospective design, which facilitated the use of meticulous and high-standard collection procedures for pre-surgical samples and allowed for a comprehensive six-month follow-up period to monitor the onset of cachexia in patients. Pre-surgical or pre-treatment fecal sampling may serve as a valuable tool for the prediction of cachexia onset in CRC patients. Gut microbiome abundances and diversities were quantified using cutting-edge standardized protocols for biospecimen collection, processing, storage, and analyses. Rigorous quality controls were integrated into our laboratory procedures to mitigate measurement errors, including batch effects, while analytical methods accounted for multiple testing. Factors potentially influencing gut microbiome composition, such as medication use and systemic inflammation, were evaluated during stool collection and further adjusted for during statistical analyses.

The study has a few limitations. Our findings require validation, as one of the a priori-selected bacteria previously associated with CRC and cachexia in the literature showed a statistically significant association while another showed a suggestive association in our study. Thus, caution is warranted in the interpretation of the study findings. There is lack of generalization of study findings to other racial and ethnic groups as the study participants were predominantly White from US (Utah) and Germany (Heidelberg) despite encompassing different geographical regions. Additionally, geographic differences in study sites may have contributed to the observed alpha-diversity patterns. In stratified analyses, we noted that German participants showed a stronger inverse association with *Porphyromonas*. These differences may reflect underlying lifestyle, dietary, and cultural factors that vary between the German and U.S. sites and are known to influence gut microbiome composition and diversity. Further investigation is warranted to understand how regional factors may shape the gut microbiome. Another potential limitation is that we were unable to assess cachexia status at baseline. Some patients may have already been cachectic at the time of surgery and subsequently experienced minimal or no additional weight loss, leading to potential misclassification as non-cachectic at six months post-surgery. Future studies should assess cachexia status at diagnosis using standardized clinical criteria to better capture its onset and progression. We used 16S rRNA gene sequencing, which cannot classify taxa at the species level or characterize functional genes. Nevertheless, 16S rRNA gene sequencing is a valuable and frequently utilized method for identifying initial microbial targets and provides a broad overview of the microbial community structure. This study is based on a single stool sample collected prior to surgery, which limits our ability to assess temporal changes in the gut microbiome. Cachexia was evaluated 6 months post-surgery, and intervening exposures such as neoadjuvant therapy, surgery, and adjuvant treatment may have influenced both host metabolism and microbial composition. The gut microbiome is known to be disrupted by these procedures [45]. To explore the potential modifying effects of treatment, we conducted stratified analyses by neoadjuvant and adjuvant treatment status. Nonetheless, these findings are hypothesis-generating and should be interpreted with caution. Future studies incorporating longitudinal stool sampling across multiple time points are needed to better characterize the dynamics of the microbiome–cachexia relationship and identify critical windows for intervention. While the present study focused on taxonomic profiling of the gut microbiome using 16S rRNA gene sequencing, we anticipate expanding this work through metatranscriptomic analyses in future analyses. Total RNA was co-isolated during sample processing and will be used in future studies to characterize microbial gene expression profiles. These functional insights will allow us to move beyond taxonomic associations and better understand the mechanistic role of the microbiome in cachexia development. Ultimately, this approach may help identify functionally relevant microbial targets for intervention. Therefore, future research should incorporate metagenomic, metabolomic, and metatranscriptomic analyses to examine associations at the species level and gain deeper insights into the functional and dynamic changes within microbial communities.

In conclusion, higher gut microbial alpha-diversity and lower relative abundances of the genera *Porphyromonas* and *Actinomyces* in pre-surgery stool samples were associated with the onset of cachexia among CRC patients six months post-surgery. Future research involving larger sample sizes is essential to confirm our findings and to investigate the roles of the identified genera at the species level. This deeper understanding will help elucidate the biological mechanisms underlying the onset of cachexia, facilitate earlier detection and contribute to the development of targeted interventions and therapeutic strategies for managing cachexia in CRC patients.

## Supplementary Information

Below is the link to the electronic supplementary material.

Supplementary file1 (DOCX 102 kb)

Supplementary file2 (DOCX 295 kb)

## Data Availability

The ColoCare Study data is available from colocarestudy_admin@hci.utah.edu on reasonable request and as described on the ColoCare website (https://uofuhealth.utah.edu/huntsman/labs/colocare-consortium/). Our data-sharing procedures are available online (https://uofuhealth.utah.edu/huntsman/labs/colocare-consortium/data-sharing/new-projects.php). For questions, please contact colocarestudy_admin@hci.utah.edu. Further information is available from the corresponding author upon request.
