# Supplementary material for: Pre-surgery gut microbial diversity and abundance are associated with post-surgery onset of cachexia in colorectal cancer patients: the ColoCare Study
Source: Cancer Causes Control. 2025 Sep 4;36(12):1795–812. doi: 10.1007/s10552-025-02042-y (PMC12478455; doi:10.1007/s10552-025-02042-y)
Supplement: Supplementary file 1 — Supplementary file1 (DOCX 102 kb) [file 10552_2025_2042_MOESM1_ESM.docx]

**Supplemental Table 1:** Associations of *a priori*-selected bacterial relative abundances with cachexia onset at 6 months post-surgery stratified by patient characteristics in the ColoCare Study, n= 103

| **Tumor site, n (%)** |  | **Colon, 48 (47)** |  | **Rectum, 55 (53)** |  |  |
| --- | --- | --- | --- | --- | --- | --- |
|  | Mean (min-max) | OR (95% CI) | P-value | OR (95% CI) | P-value | P-interaction |
| ***A priori*-selected bacteria associated with CRC** |  |  |  |  |  |  |
| *Bacteroides* | 9.30 (0.00, 64.49) | 0.62 (0.32, 1.21) | 0.16 | 0.91 (0.54, 1.52) | 0.72 | 0.12 |
| Clostridiales (Order) | 73.16 (18.47, 96.49) | 1.47 (0.46, 4.66) | 0.51 | 1.05 (0.57, 1.94) | 0.87 | 0.60 |
| *Dialister* | 0.88 (0.00, 6.39) | 1.12 (0.59, 2.12) | 0.73 | 0.88 (0.52, 1.51) | 0.65 | 0.86 |
| *Fusobacterium* | 0.18 (0.00, 10.77) | 1.23 (0.62, 2.41) | 0.56 | 0.92 (0.54, 1.55) | 0.74 | 0.72 |
| *Parvimonas* | 0.14 (0.00, 6.68) | 0.66 (0.29, 1.46) | 0.30 | 1.16 (0.62, 2.18) | 0.65 | 0.40 |
| *Peptostreptococcus* | 0.35 (0.00, 17.43) | 0.99 (0.47, 2.07) | 0.97 | 1.11 (0.69, 1.79) | 0.68 | 0.95 |
| *Porphyromonas* | 0.10 (0.00, 3.36) | 0.69 (0.09, 5.50) | 0.72 | 0.57 (0.31, 1.04) | 0.07 | 0.71 |
| *Prevotella* | 2.81 (0.00, 48.70) | 0.93 (0.46, 1.87) | 0.85 | 1.10 (0.76, 1.60) | 0.62 | 0.87 |
| ***A priori*-selected bacteria associated with cachexia in non-CRC patients** |  |  |  |  |  |  |
| *Actinomyces* | 0.05 (0.00, 0.88) | 1.20 (0.67, 2.15) | 0.53 | 0.72 (0.47, 1.10) | 0.13 | 0.79 |
| *Lactobacillus* | 0.70 (0.00, 36.87) | 1.63 (0.86, 3.07) | 0.13 | 0.80 (0.49, 1.31) | 0.38 | 0.18 |
| *Parabacteroides* | 0.89 (0.00, 4.83) | 0.53 (0.21, 1.34) | 0.18 | 1.15 (0.57, 2.34) | 0.69 | 0.30 |
| *Bacteroides* | 9.30 (0.00, 64.49) | 0.62 (0.32, 1.21) | 0.16 | 0.91 (0.54, 1.52) | 0.72 | 0.12 |
| *Veillonella* | 0.18 (0.00, 5.66) | 0.83 (0.44, 1.56) | 0.56 | 0.84 (0.48, 1.46) | 0.54 | 0.54 |
| *Megamonas* | 0.17 (0.00, 22.30) | Inf (0.00, Inf) | 1.00 | 0.36 (0.06, 2.24) | 0.28 | 0.99 |
| *Peptococcus* | 0.06 (0.00, 1.73) | 1.14 (0.27, 4.86) | 0.86 | 1.14 (0.30, 4.36) | 0.84 | 0.86 |
| *Bifidobacterium* | 4.12 (0.00, 27.43) | 1.32 (0.71, 2.46) | 0.38 | 1.19 (0.82, 1.74) | 0.36 | 0.42 |
| *Faecalibacterium* | 8.89 (0.00, 31.58) | 1.42 (0.87, 2.32) | 0.16 | 1.22 (0.66, 2.26) | 0.53 | 0.40 |
| *Prevotella* | 2.81 (0.00, 48.70) | 0.93 (0.46, 1.87) | 0.85 | 1.10 (0.76, 1.60) | 0.62 | 0.87 |
| *Escherichia* | 0.06 (0.00, 4.86) | 0.01 (0.00, Inf) | 1.00 | Inf (0.00, Inf) | 0.99 | 0.56 |
| **Stage, n (%)** |  | **Stage I-II, 56 (54)** |  | **Stage III, 47 (46)** |  |  |
|  | Mean (min-max) | OR (95% CI) | P-value | OR (95% CI) | P-value | P-interaction |
| ***A priori*-selected bacteria associated with CRC** |  |  |  |  |  |  |
| *Bacteroides* | 9.30 (0.00, 64.49) | 0.67 (0.38, 1.20) | 0.18 | 0.70 (0.32, 1.54) | 0.38 | 0.32 |
| Clostridiales (Order) | 73.16 (18.47, 96.49) | 1.09 (0.51, 2.32) | 0.82 | 0.85 (0.33, 2.20) | 0.74 | 0.91 |
| *Dialister* | 0.88 (0.00, 6.39) | 1.28 (0.62, 2.64) | 0.50 | 0.92 (0.56, 1.50) | 0.73 | 0.76 |
| *Fusobacterium* | 0.18 (0.00, 10.77) | 0.95 (0.50, 1.82) | 0.89 | 1.29 (0.75, 2.23) | 0.35 | 0.77 |
| *Parvimonas* | 0.14 (0.00, 6.68) | 1.14 (0.53, 2.44) | 0.74 | 1.02 (0.52, 2.00) | 0.95 | 0.87 |
| *Peptostreptococcus* | 0.35 (0.00, 17.43) | 1.22 (0.72, 2.07) | 0.47 | 1.19 (0.61, 2.29) | 0.61 | 0.66 |
| *Porphyromonas* | 0.10 (0.00, 3.36) | 0.73 (0.29, 1.81) | 0.50 | 0.41 (0.15, 1.12) | 0.08 | 0.46 |
| *Prevotella* | 2.81 (0.00, 48.70) | 0.84 (0.46, 1.53) | 0.56 | 1.12 (0.72, 1.76) | 0.61 | 0.70 |
| ***A priori*-selected bacteria associated with cachexia in non-CRC patients** |  |  |  |  |  |  |
| *Actinomyces* | 0.05 (0.00, 0.88) | 1.00 (0.60, 1.65) | 0.99 | 0.84 (0.50, 1.42) | 0.51 | 0.54 |
| *Lactobacillus* | 0.70 (0.00, 36.87) | 0.98 (0.62, 1.56) | 0.94 | 1.26 (0.59, 2.68) | 0.55 | 0.87 |
| *Parabacteroides* | 0.89 (0.00, 4.83) | 0.84 (0.38, 1.90) | 0.68 | 0.72 (0.31, 1.65) | 0.44 | 0.80 |
| *Bacteroides* | 9.30 (0.00, 64.49) | 0.67 (0.38, 1.20) | 0.18 | 0.70 (0.32, 1.54) | 0.38 | 0.32 |
| *Veillonella* | 0.18 (0.00, 5.66) | 0.64 (0.35, 1.16) | 0.14 | 0.87 (0.40, 1.87) | 0.71 | 0.18 |
| *Megamonas* | 0.17 (0.00, 22.30) | Inf (0.00, Inf) | 0.99 | 0.76 (0.13, 4.48) | 0.76 | 0.58 |
| *Peptococcus* | 0.06 (0.00, 1.73) | 1.85 (0.40, 8.50) | 0.43 | 1.66 (0.42, 6.65) | 0.47 | 0.81 |
| *Bifidobacterium* | 4.12 (0.00, 27.43) | 1.24 (0.78, 1.99) | 0.37 | 1.30 (0.79, 2.14) | 0.31 | 0.59 |
| *Faecalibacterium* | 8.89 (0.00, 31.58) | 1.50 (0.88, 2.56) | 0.14 | 0.92 (0.55, 1.56) | 0.76 | 0.20 |
| *Prevotella* | 2.81 (0.00, 48.70) | 0.84 (0.46, 1.53) | 0.56 | 1.12 (0.72, 1.76) | 0.61 | 0.70 |
| *Escherichia* | 0.06 (0.00, 4.86) | 1.08 (0.39, 2.98) | 0.88 | Inf (0.00, Inf) | 1.00 | 0.72 |
| **Sex, n (%)** |  | **Male, 54 (52)** |  | **Female, 49 (48)** |  |  |
|  | Mean (min-max) | OR (95% CI) | P-value | OR (95% CI) | P-value | P-interaction |
| ***A priori*-selected bacteria associated with CRC** |  |  |  |  |  |  |
| *Bacteroides* | 9.30 (0.00, 64.49) | 1.00 (0.59, 1.70) | 1.00 | 0.59 (0.32, 1.09) | 0.09 | 0.14 |
| Clostridiales (Order) | 73.16 (18.47, 96.49) | 1.36 (0.61, 3.03) | 0.45 | 0.92 (0.38, 2.21) | 0.85 | 0.77 |
| *Dialister* | 0.88 (0.00, 6.39) | 0.78 (0.48, 1.27) | 0.32 | 1.40 (0.66, 2.97) | 0.38 | 0.30 |
| *Fusobacterium* | 0.18 (0.00, 10.77) | 0.75 (0.44, 1.28) | 0.29 | 1.98 (0.87, 4.50) | 0.10 | 0.12 |
| *Parvimonas* | 0.14 (0.00, 6.68) | 1.26 (0.48, 3.30) | 0.64 | 0.93 (0.50, 1.72) | 0.82 | 0.75 |
| *Peptostreptococcus* | 0.35 (0.00, 17.43) | 1.22 (0.70, 2.13) | 0.49 | 0.93 (0.49, 1.79) | 0.84 | 0.65 |
| *Porphyromonas* | 0.10 (0.00, 3.36) | 0.34 (0.12, 1.00) | 0.05 | 0.65 (0.29, 1.43) | 0.28 | 0.37 |
| *Prevotella* | 2.81 (0.00, 48.70) | 1.15 (0.77, 1.70) | 0.50 | 1.05 (0.56, 1.97) | 0.88 | 0.66 |
| ***A priori*-selected bacteria associated with cachexia in non-CRC patients** |  |  |  |  |  |  |
| *Actinomyces* | 0.05 (0.00, 0.88) | 1.07 (0.65, 1.74) | 0.80 | 0.70 (0.42, 1.17) | 0.17 | 0.08 |
| *Lactobacillus* | 0.70 (0.00, 36.87) | 0.63 (0.34, 1.17) | 0.14 | 1.59 (0.88, 2.89) | 0.12 | 0.15 |
| *Parabacteroides* | 0.89 (0.00, 4.83) | 1.23 (0.63, 2.37) | 0.54 | 0.76 (0.34, 1.67) | 0.49 | 0.53 |
| *Bacteroides* | 9.30 (0.00, 64.49) | 1.00 (0.59, 1.70) | 1.00 | 0.59 (0.32, 1.09) | 0.09 | 0.14 |
| *Veillonella* | 0.18 (0.00, 5.66) | 0.61 (0.27, 1.36) | 0.23 | 0.96 (0.57, 1.64) | 0.89 | 0.83 |
| *Megamonas* | 0.17 (0.00, 22.30) | 0.31 (0.01, 11.04) | 0.52 | Inf (0.00, Inf) | 1.00 | 0.99 |
| *Peptococcus* | 0.06 (0.00, 1.73) | 0.49 (0.08, 3.20) | 0.46 | 1.70 (0.41, 7.03) | 0.46 | 0.65 |
| *Bifidobacterium* | 4.12 (0.00, 27.43) | 1.06 (0.72, 1.57) | 0.76 | 2.01 (1.03, 3.92) | 0.04 | 0.09 |
| *Faecalibacterium* | 8.89 (0.00, 31.58) | 1.58 (0.75, 3.33) | 0.23 | 1.08 (0.71, 1.66) | 0.71 | 0.61 |
| *Prevotella* | 2.81 (0.00, 48.70) | 1.15 (0.77, 1.70) | 0.50 | 1.05 (0.56, 1.97) | 0.88 | 0.66 |
| *Escherichia* | 0.06 (0.00, 4.86) | 1.18 (0.46, 3.03) | 0.73 | Inf (0.00, Inf) | 0.99 | 0.99 |
| **Age, years old, n (%)** |  | **<65, 60 (58)** |  | **≥ 65, 43 (42)** |  |  |
|  | Mean (min-max) | OR (95% CI) | P-value | OR (95% CI) | P-value | P-interaction |
| ***A priori*-selected bacteria associated with CRC** |  |  |  |  |  |  |
| *Bacteroides* | 9.30 (0.00, 64.49) | 0.83 (0.51, 1.36) | 0.46 | 0.78 (0.39, 1.56) | 0.49 | 0.57 |
| Clostridiales (Order) | 73.16 (18.47, 96.49) | 1.19 (0.65, 2.17) | 0.58 | 1.91 (0.39, 9.39) | 0.42 | 0.89 |
| *Dialister* | 0.88 (0.00, 6.39) | 0.90 (0.56, 1.45) | 0.67 | 2.01 (0.79, 5.10) | 0.14 | 0.58 |
| *Fusobacterium* | 0.18 (0.00, 10.77) | 1.12 (0.65, 1.93) | 0.67 | 0.79 (0.32, 1.95) | 0.60 | 0.40 |
| *Parvimonas* | 0.14 (0.00, 6.68) | 1.14 (0.63, 2.07) | 0.66 | 0.59 (0.18, 1.91) | 0.38 | 0.59 |
| *Peptostreptococcus* | 0.35 (0.00, 17.43) | 1.10 (0.69, 1.75) | 0.70 | 0.74 (0.31, 1.80) | 0.51 | 0.65 |
| *Porphyromonas* | 0.10 (0.00, 3.36) | 0.67 (0.35, 1.27) | 0.22 | 0.40 (0.09, 1.75) | 0.22 | 0.14 |
| *Prevotella* | 2.81 (0.00, 48.70) | 1.11 (0.77, 1.61) | 0.58 | 0.82 (0.35, 1.91) | 0.64 | 0.72 |
| ***A priori*-selected bacteria associated with cachexia in non-CRC patients** |  |  |  |  |  |  |
| *Actinomyces* | 0.05 (0.00, 0.88) | 0.67 (0.42, 1.05) | 0.08 | 1.22 (0.69, 2.16) | 0.49 | 0.06 |
| *Lactobacillus* | 0.70 (0.00, 36.87) | 0.94 (0.59, 1.50) | 0.80 | 0.95 (0.51, 1.75) | 0.86 | 0.91 |
| *Parabacteroides* | 0.89 (0.00, 4.83) | 1.11 (0.53, 2.30) | 0.78 | 0.61 (0.24, 1.51) | 0.28 | 0.45 |
| *Bacteroides* | 9.30 (0.00, 64.49) | 0.83 (0.51, 1.36) | 0.46 | 0.78 (0.39, 1.56) | 0.49 | 0.57 |
| *Veillonella* | 0.18 (0.00, 5.66) | 2.07 (0.86, 4.98) | 0.10 | 0.48 (0.24, 0.98) | 0.04 | 0.18 |
| *Megamonas* | 0.17 (0.00, 22.30) | 0.40 (0.02, 7.10) | 0.53 | 0.01 (0.00, Inf) | 1.00 | 0.58 |
| *Peptococcus* | 0.06 (0.00, 1.73) | 1.30 (0.37, 4.53) | 0.68 | 0.43 (0.02, 7.68) | 0.57 | 0.67 |
| *Bifidobacterium* | 4.12 (0.00, 27.43) | 1.41 (0.91, 2.18) | 0.13 | 0.79 (0.44, 1.42) | 0.44 | 0.13 |
| *Faecalibacterium* | 8.89 (0.00, 31.58) | 1.35 (0.77, 2.35) | 0.29 | 1.46 (0.84, 2.54) | 0.18 | 0.30 |
| *Prevotella* | 2.81 (0.00, 48.70) | 1.11 (0.77, 1.61) | 0.58 | 0.82 (0.35, 1.91) | 0.64 | 0.72 |
| *Escherichia* | 0.06 (0.00, 4.86) | Inf (0.00, Inf) | 0.99 | 1.42 (0.49, 4.17) | 0.52 | 0.99 |
| **Adjuvant Treatment,**  **n (%)** |  | **No, 64 (62)** |  | **Yes, 39 (38)** |  |  |
|  | Mean (min-max) | OR (95% CI) | P-value | OR (95% CI) | P-value | P-interaction |
| ***A priori*-selected bacteria associated with CRC** |  |  |  |  |  |  |
| *Bacteroides* | 9.30 (0.00, 64.49) | 0.81 (0.49, 1.35) | 0.42 | 0.47 (0.19, 1.17) | 0.11 | 0.50 |
| Clostridiales (Order) | 73.16 (18.47, 96.49) | 0.89 (0.42, 1.85) | 0.75 | 1.19 (0.44, 3.18) | 0.73 | 0.71 |
| *Dialister* | 0.88 (0.00, 6.39) | 0.97 (0.52, 1.81) | 0.93 | 1.04 (0.62, 1.76) | 0.88 | 0.71 |
| *Fusobacterium* | 0.18 (0.00, 10.77) | 0.79 (0.43, 1.45) | 0.44 | 1.47 (0.75, 2.88) | 0.26 | 0.29 |
| *Parvimonas* | 0.14 (0.00, 6.68) | 0.62 (0.25, 1.50) | 0.29 | 1.27 (0.66, 2.44) | 0.48 | 0.45 |
| *Peptostreptococcus* | 0.35 (0.00, 17.43) | 0.93 (0.53, 1.64) | 0.82 | 1.38 (0.70, 2.71) | 0.35 | 0.76 |
| *Porphyromonas* | 0.10 (0.00, 3.36) | 0.47 (0.15, 1.49) | 0.20 | 0.48 (0.18, 1.28) | 0.14 | 0.27 |
| *Prevotella* | 2.81 (0.00, 48.70) | 0.94 (0.54, 1.61) | 0.81 | 1.13 (0.69, 1.87) | 0.62 | 0.91 |
| ***A priori*-selected bacteria associated with cachexia in non-CRC patients** |  |  |  |  |  |  |
| *Actinomyces* | 0.05 (0.00, 0.88) | 0.87 (0.52, 1.45) | 0.59 | 0.95 (0.52, 1.73) | 0.87 | 0.46 |
| *Lactobacillus* | 0.70 (0.00, 36.87) | 0.97 (0.61, 1.54) | 0.88 | 1.56 (0.64, 3.79) | 0.33 | 0.90 |
| *Parabacteroides* | 0.89 (0.00, 4.83) | 1.21 (0.53, 2.74) | 0.65 | 0.52 (0.20, 1.37) | 0.19 | 0.74 |
| *Bacteroides* | 9.30 (0.00, 64.49) | 0.81 (0.49, 1.35) | 0.42 | 0.47 (0.19, 1.17) | 0.11 | 0.50 |
| *Veillonella* | 0.18 (0.00, 5.66) | 0.58 (0.31, 1.10) | 0.09 | 0.93 (0.44, 1.99) | 0.86 | 0.13 |
| *Megamonas* | 0.17 (0.00, 22.30) | Inf (0.00, Inf) | 0.99 | 0.00 (0.00, Inf) | 1.00 | 0.55 |
| *Peptococcus* | 0.06 (0.00, 1.73) | 0.40 (0.04, 4.35) | 0.45 | 2.17 (0.38, 12.49) | 0.39 | 0.36 |
| *Bifidobacterium* | 4.12 (0.00, 27.43) | 1.36 (0.85, 2.17) | 0.20 | 1.14 (0.71, 1.85) | 0.58 | 0.41 |
| *Faecalibacterium* | 8.89 (0.00, 31.58) | 1.45 (0.87, 2.42) | 0.15 | 0.90 (0.55, 1.47) | 0.68 | 0.17 |
| *Prevotella* | 2.81 (0.00, 48.70) | 0.94 (0.54, 1.61) | 0.81 | 1.13 (0.69, 1.87) | 0.62 | 0.91 |
| *Escherichia* | 0.06 (0.00, 4.86) | 1.10 (0.41, 2.97) | 0.85 | Inf (0.00, Inf) | 1.00 | 0.74 |
| **Neo-adjuvant Treatment,**  **n (%)** |  | **No, 76 (74)** |  | **Yes, 27 (26)** |  |  |
|  | Mean (min-max) | OR (95% CI) | P-value | OR (95% CI) | P-value | P-interaction |
| ***A priori*-selected bacteria associated with CRC** |  |  |  |  |  |  |
| *Bacteroides* | 9.30 (0.00, 64.49) | 0.93 (0.58, 1.49) | 0.76 | 0.35 (0.10, 1.28) | 0.11 | 0.44 |
| Clostridiales (Order) | 73.16 (18.47, 96.49) | 1.33 (0.59, 2.98) | 0.49 | 1.45 (0.44, 4.73) | 0.54 | 0.55 |
| *Dialister* | 0.88 (0.00, 6.39) | 1.07 (0.62, 1.84) | 0.81 | 1.29 (0.57, 2.95) | 0.54 | 0.70 |
| *Fusobacterium* | 0.18 (0.00, 10.77) | 0.88 (0.52, 1.50) | 0.65 | 1.02 (0.31, 3.33) | 0.98 | 0.78 |
| *Parvimonas* | 0.14 (0.00, 6.68) | 1.08 (0.60, 1.96) | 0.79 | 0.18 (0.01, 4.55) | 0.30 | 0.56 |
| *Peptostreptococcus* | 0.35 (0.00, 17.43) | 1.00 (0.60, 1.67) | 1.00 | 1.05 (0.43, 2.60) | 0.91 | 0.70 |
| *Porphyromonas* | 0.10 (0.00, 3.36) | 1.02 (0.35, 3.01) | 0.97 | 0.36 (0.12, 1.04) | 0.06 | 0.69 |
| *Prevotella* | 2.81 (0.00, 48.70) | 1.20 (0.76, 1.92) | 0.44 | 0.73 (0.33, 1.60) | 0.43 | 0.26 |
| ***A priori*-selected bacteria associated with cachexia in non-CRC patients** |  |  |  |  |  |  |
| *Actinomyces* | 0.05 (0.00, 0.88) | 0.85 (0.55, 1.31) | 0.46 | 0.51 (0.21, 1.20) | 0.12 | 0.63 |
| *Lactobacillus* | 0.70 (0.00, 36.87) | 0.97 (0.62, 1.52) | 0.90 | 1.16 (0.36, 3.70) | 0.81 | 0.72 |
| *Parabacteroides* | 0.89 (0.00, 4.83) | 0.91 (0.48, 1.74) | 0.78 | 0.61 (0.16, 2.26) | 0.46 | 0.68 |
| *Bacteroides* | 9.30 (0.00, 64.49) | 0.93 (0.58, 1.49) | 0.76 | 0.35 (0.10, 1.28) | 0.11 | 0.44 |
| *Veillonella* | 0.18 (0.00, 5.66) | 0.63 (0.36, 1.12) | 0.12 | 0.45 (0.15, 1.38) | 0.16 | 0.28 |
| *Megamonas* | 0.17 (0.00, 22.30) | 0.54 (0.01, 21.80) | 0.75 | 0.47 (0.06, 3.64) | 0.47 | 0.56 |
| *Peptococcus* | 0.06 (0.00, 1.73) | 0.67 (0.23, 1.99) | 0.47 | 1.56 (0.14, 17.38) | 0.72 | 0.69 |
| *Bifidobacterium* | 4.12 (0.00, 27.43) | 1.07 (0.70, 1.63) | 0.76 | 1.73 (0.92, 3.24) | 0.09 | 0.76 |
| *Faecalibacterium* | 8.89 (0.00, 31.58) | 1.27 (0.84, 1.93) | 0.26 | 0.93 (0.34, 2.58) | 0.90 | 0.33 |
| *Prevotella* | 2.81 (0.00, 48.70) | 1.20 (0.76, 1.92) | 0.44 | 0.73 (0.33, 1.60) | 0.43 | 0.26 |
| *Escherichia* | 0.06 (0.00, 4.86) | 1.07 (0.45, 2.54) | 0.87 | Inf (0.00, Inf) | 1.00 | 0.66 |
| **Physical Activity,**  **MET hrs/week, n (%)** |  | **≥8.75, 43 (42)** |  | **<8.75, 60 (58)** |  |  |
|  | Mean (min-max) | OR (95% CI) | P-value | OR (95% CI) | P-value | P-interaction |
| ***A priori*-selected bacteria associated with CRC** |  |  |  |  |  |  |
| *Bacteroides* | 9.30 (0.00, 64.49) | 1.12 (0.59, 2.13) | 0.74 | 0.64 (0.37, 1.13) | 0.13 | 0.93 |
| Clostridiales (Order) | 73.16 (18.47, 96.49) | 0.79 (0.32, 1.93) | 0.60 | 1.31 (0.61, 2.80) | 0.49 | 0.62 |
| *Dialister* | 0.88 (0.00, 6.39) | 0.91 (0.49, 1.68) | 0.76 | 0.73 (0.39, 1.39) | 0.34 | 1.00 |
| *Fusobacterium* | 0.18 (0.00, 10.77) | 0.90 (0.48, 1.69) | 0.74 | 0.91 (0.50, 1.67) | 0.76 | 0.81 |
| *Parvimonas* | 0.14 (0.00, 6.68) | 0.92 (0.44, 1.93) | 0.82 | 0.65 (0.29, 1.47) | 0.30 | 0.96 |
| *Peptostreptococcus* | 0.35 (0.00, 17.43) | 1.66 (0.83, 3.33) | 0.15 | 0.64 (0.33, 1.25) | 0.19 | 0.08 |
| *Porphyromonas* | 0.10 (0.00, 3.36) | 0.40 (0.13, 1.21) | 0.11 | 0.47 (0.20, 1.09) | 0.08 | 0.24 |
| *Prevotella* | 2.81 (0.00, 48.70) | 0.84 (0.47, 1.50) | 0.55 | 1.16 (0.73, 1.84) | 0.52 | 0.81 |
| ***A priori*-selected bacteria associated with cachexia in non-CRC patients** |  |  |  |  |  |  |
| *Actinomyces* | 0.05 (0.00, 0.88) | 0.81 (0.46, 1.44) | 0.47 | 0.99 (0.60, 1.62) | 0.96 | 0.26 |
| *Lactobacillus* | 0.70 (0.00, 36.87) | 0.71 (0.35, 1.45) | 0.35 | 1.25 (0.78, 2.01) | 0.36 | 0.43 |
| *Parabacteroides* | 0.89 (0.00, 4.83) | 1.13 (0.49, 2.64) | 0.77 | 0.86 (0.43, 1.72) | 0.67 | 0.84 |
| *Bacteroides* | 9.30 (0.00, 64.49) | 1.12 (0.59, 2.13) | 0.74 | 0.64 (0.37, 1.13) | 0.13 | 0.93 |
| *Veillonella* | 0.18 (0.00, 5.66) | 0.66 (0.30, 1.43) | 0.29 | 0.95 (0.50, 1.79) | 0.87 | 0.29 |
| *Megamonas* | 0.17 (0.00, 22.30) | 0.54 (0.06, 5.04) | 0.59 | 0.01 (0.00, Inf) | 1.00 | 0.67 |
| *Peptococcus* | 0.06 (0.00, 1.73) | 0.63 (0.09, 4.47) | 0.64 | 0.83 (0.21, 3.25) | 0.79 | 0.92 |
| *Bifidobacterium* | 4.12 (0.00, 27.43) | 0.77 (0.38, 1.58) | 0.48 | 1.41 (0.96, 2.06) | 0.08 | 0.65 |
| *Faecalibacterium* | 8.89 (0.00, 31.58) | 0.75 (0.41, 1.37) | 0.35 | 1.86 (1.06, 3.25) | 0.03 | 0.32 |
| *Prevotella* | 2.81 (0.00, 48.70) | 0.84 (0.47, 1.50) | 0.55 | 1.16 (0.73, 1.84) | 0.52 | 0.81 |
| *Escherichia* | 0.06 (0.00, 4.86) | 0.91 (0.38, 2.20) | 0.83 | Inf (0.00, Inf) | 0.99 | 0.75 |
| **Dietary Fiber Intake, grams/day, n (%)** |  | **Low Fiber, 60 (70)** |  | **High Fiber, 26 (30)** |  |  |
|  | Mean (min-max) | OR (95% CI) | P-value | OR (95% CI) | P-value | P-interaction |
| ***A priori*-selected bacteria associated with CRC** |  |  |  |  |  |  |
| *Bacteroides* | 9.30 (0.00, 64.49) | 0.78 (0.40, 1.53) | 0.48 | 0.80 (0.47, 1.38) | 0.43 | 0.37 |
| Clostridiales (Order) | 73.16 (18.47, 96.49) | 0.73 (0.30, 1.75) | 0.48 | 1.58 (0.48, 5.18) | 0.45 | 0.47 |
| *Dialister* | 0.88 (0.00, 6.39) | 1.18 (0.41, 3.39) | 0.76 | 3.13 (0.84, 11.71) | 0.09 | 0.68 |
| *Fusobacterium* | 0.18 (0.00, 10.77) | 1.17 (0.70, 1.96) | 0.55 | 0.76 (0.41, 1.43) | 0.40 | 0.02 |
| *Parvimonas* | 0.14 (0.00, 6.68) | 2.22 (0.68, 7.25) | 0.19 | 0.58 (0.27, 1.27) | 0.18 | 0.24 |
| *Peptostreptococcus* | 0.35 (0.00, 17.43) | 1.77 (0.75, 4.15) | 0.19 | 0.68 (0.31, 1.46) | 0.32 | 0.28 |
| *Porphyromonas* | 0.10 (0.00, 3.36) | 0.43 (0.17, 1.06) | 0.07 | Inf (0.00, Inf) | 0.99 | 0.19 |
| *Prevotella* | 2.81 (0.00, 48.70) | 1.34 (0.55, 3.30) | 0.52 | 1.43 (0.81, 2.55) | 0.22 | 0.77 |
| ***A priori*-selected bacteria associated with cachexia in non-CRC patients** |  |  |  |  |  |  |
| *Actinomyces* | 0.05 (0.00, 0.88) | 0.86 (0.43, 1.70) | 0.66 | 0.99 (0.56, 1.73) | 0.96 | 0.40 |
| *Lactobacillus* | 0.70 (0.00, 36.87) | 1.04 (0.48, 2.27) | 0.91 | 1.29 (0.68, 2.42) | 0.44 | 0.64 |
| *Parabacteroides* | 0.89 (0.00, 4.83) | 0.98 (0.37, 2.63) | 0.97 | 0.94 (0.46, 1.95) | 0.87 | 0.90 |
| *Bacteroides* | 9.30 (0.00, 64.49) | 0.78 (0.40, 1.53) | 0.48 | 0.80 (0.47, 1.38) | 0.43 | 0.37 |
| *Veillonella* | 0.18 (0.00, 5.66) | 0.58 (0.25, 1.37) | 0.22 | 0.83 (0.45, 1.53) | 0.55 | 0.25 |
| *Megamonas* | 0.17 (0.00, 22.30) | 1.22 (0.25, 5.99) | 0.80 | NA (NA, NA) | 0.37 | 0.99 |
| *Peptococcus* | 0.06 (0.00, 1.73) | Inf (0.00, Inf) | 0.99 | 0.00 (0.00, Inf) | 1.00 | 0.98 |
| *Bifidobacterium* | 4.12 (0.00, 27.43) | 0.93 (0.50, 1.73) | 0.82 | 1.42 (0.85, 2.39) | 0.18 | 0.71 |
| *Faecalibacterium* | 8.89 (0.00, 31.58) | 0.62 (0.29, 1.31) | 0.21 | 2.89 (0.76, 10.94) | 0.12 | 0.29 |
| *Prevotella* | 2.81 (0.00, 48.70) | 1.34 (0.55, 3.30) | 0.52 | 1.43 (0.81, 2.55) | 0.22 | 0.77 |
| *Escherichia* | 0.06 (0.00, 4.86) | Inf (0.00, Inf)1 | 1.00 | 1.32 (0.43, 4.01) | 0.62 | 0.99 |
| **Energy Intake,**  **kcal, n (%)** |  | **Group 1, 32 (37)** |  | **Group 2, 54 (63)** |  |  |
|  | Mean (min-max) | OR (95% CI) | P-value | OR (95% CI) | P-value | P-interaction |
| ***A priori*-selected bacteria associated with CRC** |  |  |  |  |  |  |
| *Bacteroides* | 9.30 (0.00, 64.49) | 0.98 (0.42, 2.28) | 0.96 | 0.80 (0.47, 1.38) | 0.43 | 0.62 |
| Clostridiales (Order) | 73.16 (18.47, 96.49) | 1.26 (0.45, 3.55) | 0.66 | 1.58 (0.48, 5.18) | 0.45 | 0.74 |
| *Dialister* | 0.88 (0.00, 6.39) | 3.59 (0.65, 19.95) | 0.14 | 0.79 (0.45, 1.39) | 0.41 | 0.34 |
| *Fusobacterium* | 0.18 (0.00, 10.77) | 0.96 (0.57, 1.61) | 0.88 | 1.55 (0.63, 3.86) | 0.34 | 0.49 |
| *Parvimonas* | 0.14 (0.00, 6.68) | 0.66 (0.17, 2.55) | 0.55 | 0.58 (0.27, 1.27) | 0.18 | 0.70 |
| *Peptostreptococcus* | 0.35 (0.00, 17.43) | 2.01 (0.67, 6.02) | 0.21 | 1.15 (0.63, 2.12) | 0.64 | 0.91 |
| *Porphyromonas* | 0.10 (0.00, 3.36) | 0.68 (0.16, 2.92) | 0.60 | 0.62 (0.17, 2.27) | 0.47 | 0.98 |
| *Prevotella* | 2.81 (0.00, 48.70) | 0.41 (0.14, 1.27) | 0.12 | 1.43 (0.81, 2.55) | 0.22 | 0.27 |
| ***A priori*-selected bacteria associated with cachexia in non-CRC patients** |  |  |  |  |  |  |
| *Actinomyces* | 0.05 (0.00, 0.88) | 0.54 (0.24, 1.23) | 0.14 | 0.99 (0.56, 1.73) | 0.96 | 0.25 |
| *Lactobacillus* | 0.70 (0.00, 36.87) | 0.90 (0.45, 1.08) | 0.76 | 1.29 (0.68, 2.42) | 0.44 | 0.46 |
| *Parabacteroides* | 0.89 (0.00, 4.83) | 0.69 (0.26, 1.81) | 0.45 | 1.17 (0.55, 2.49) | 0.68 | 0.93 |
| *Bacteroides* | 9.30 (0.00, 64.49) | 0.69 (0.36, 1.35) | 0.28 | 0.80 (0.47, 1.38) | 0.43 | 0.62 |
| *Veillonella* | 0.18 (0.00, 5.66) | 0.35 (0.12, 1.00) | 0.05 | 0.83 (0.45, 1.53) | 0.55 | 0.14 |
| *Megamonas* | 0.17 (0.00, 22.30) | 3.27 (0.00, Inf) | 1.00 | 0.40 (0.00, 88.04) | 0.74 | 0.99 |
| *Peptococcus* | 0.06 (0.00, 1.73) | 0.25 (0.01, 6.35) | 0.40 | 0.00 (0.00, Inf) | 1.00 | 0.99 |
| *Bifidobacterium* | 4.12 (0.00, 27.43) | 1.76 (0.83, 3.71) | 0.14 | 1.42 (0.85, 2.39) | 0.18 | 0.34 |
| *Faecalibacterium* | 8.89 (0.00, 31.58) | 2.52 (0.82, 7.75) | 0.11 | 1.46 (0.90, 2.37) | 0.13 | 0.65 |
| *Prevotella* | 2.81 (0.00, 48.70) | 0.89 (0.36, 2.20) | 0.80 | 1.43 (0.81, 2.55) | 0.22 | 0.27 |
| *Escherichia* | 0.06 (0.00, 4.86) | Inf (0.00, Inf) | 1.00 | 1.32 (0.43, 4.01) | 0.62 | 0.99 |

Adjusted for baseline age (<65 years old, ≥65 years old), sex (male or female), stage at diagnosis (I-II, III), tumor site (colon or rectum), smoking status (current, former, never), recruitment center (Heidelberg University Hospital, Germany or Huntsman Cancer Institute, Salt Lake City)

*Abbreviations*: OR, Odds Ratios; CI, Confidence Intervals

**Supplemental Table 2:** Associations of alpha-diversity with cachexia onset at 6 months post-surgery stratified by recruitment center in the ColoCare Study, n= 103

| **Study site, n (%)** | **HCI, 30 (29)** |  | **HD, 73 (71)** |  |  |
| --- | --- | --- | --- | --- | --- |
|  | OR (95% CI) | P-value | OR (95% CI) | P-value | P-interaction |
| **Alpha diversity metrics** |  |  |  |  |  |
| Shannon index | 2.14 (0.49, 9.40) | 0.32 | 1.92 (0.86, 4.29) | 0.11 | 0.23 |
| Observed Species | 1.60 (0.48, 5.31) | 0.45 | 1.57 (0.83, 2.96) | 0.17 | 0.35 |
| Faith’s PD | 1.50 (0.50, 4.51) | 0.47 | 1.65 (0.85, 3.22) | 0.14 | 0.39 |

Alpha diversity metrics were standardized to a mean of 0 and standard deviation of 1. Standardized alpha-diversity Z-scores were estimated: Z = (X-μ)/σ, where Z = Z-score, X =observed alpha-diversity value for a specific sample or patient, μ = average alpha-diversity value across all samples or the mean of the entire patient distribution, and σ = standard deviation of the alpha-diversity values across all samples or the entire patient distribution.

Adjusted for baseline age (<65 years old, ≥65 years old), sex (male or female), stage at diagnosis (I-II, III), tumor site (colon or rectum), smoking status (current, former, never), recruitment center (Heidelberg University Hospital, Germany or Huntsman Cancer Institute, Salt Lake City)

*Abbreviations*: OR, Odds Ratios; CI, Confidence Intervals; HCI, Huntsman Cancer Institute; HD, Heidelberg

.

**Supplemental Table 3:** Associations of *a priori*-selected bacterial relative abundances with cachexia onset at 6 months post-surgery stratified by recruitment center in the ColoCare Study, n= 103

| **Study site, n (%)** |  | **HCI, 30 (29)** |  | **HD, 73 (71)** |  |  |
| --- | --- | --- | --- | --- | --- | --- |
|  | Mean (min-max) | OR (95% CI) | P-value | OR (95% CI) | P-value | P-interaction |
| ***A priori*-selected bacteria associated with CRC** |  |  |  |  |  |  |
| *Bacteroides* | 9.30 (0.00, 64.49) | 0.16 (0.01, 1.88) | 0.14 | 1.01 (0.63, 1.62) | 0.97 | 0.14 |
| Clostridiales (Order) | 73.16 (18.47, 96.49) | 1.16 (0.36, 3.79) | 0.80 | 1.37 (0.60, 3.13) | 0.45 | 0.84 |
| *Dialister* | 0.88 (0.00, 6.39) | 3.64 (0.58, 23.00) | 0.17 | 0.82 (0.52, 1.30) | 0.41 | 0.37 |
| *Fusobacterium* | 0.18 (0.00, 10.77) | 0.71 (0.27, 1.84) | 0.48 | 1.14 (0.69, 1.89) | 0.60 | 0.62 |
| *Parvimonas* | 0.14 (0.00, 6.68) | 0.46 (0.09, 2.34) | 0.35 | 0.95 (0.56, 1.62) | 0.85 | 0.48 |
| *Peptostreptococcus* | 0.35 (0.00, 17.43) | 1.01 (0.38, 2.73) | 0.98 | 1.10 (0.68, 1.76) | 0.70 | 0.59 |
| *Porphyromonas* | 0.10 (0.00, 3.36) | 0.71 (0.17, 3.02) | 0.65 | 0.46 (0.22, 0.98) | **0.04** | 0.58 |
| *Prevotella* | 2.81 (0.00, 48.70) | 0.37 (0.11, 1.29) | 0.12 | 1.20 (0.84, 1.74) | 0.32 | 0.14 |
| ***A priori*-selected bacteria associated with cachexia in non-CRC patients** |  |  |  |  |  |  |
| *Actinomyces* | 0.05 (0.00, 0.88) | 0.92 (0.38, 2.23) | 0.85 | 0.76 (0.52, 1.12) | 0.17 | 0.91 |
| *Lactobacillus* | 0.70 (0.00, 36.87) | 1.03 (0.45, 2.35) | 0.95 | 0.91 (0.58, 1.41) | 0.66 | 0.79 |
| *Parabacteroides* | 0.89 (0.00, 4.83) | 0.15 (0.02, 1.20) | 0.07 | 1.08 (0.63, 1.87) | 0.77 | 0.59 |
| *Bacteroides* | 9.30 (0.00, 64.49) | 0.16 (0.01, 1.88) | 0.14 | 1.01 (0.63, 1.62) | 0.97 | 0.14 |
| *Veillonella* | 0.18 (0.00, 5.66) | 2.49 (0.55, 11.25) | 0.24 | 0.63 (0.37, 1.07) | 0.09 | 0.49 |
| *Megamonas* | 0.17 (0.00, 22.30) | Inf (0.00, Inf) | 1.00 | 0.62 (0.14, 2.80) | 0.53 | 0.99 |
| *Peptococcus* | 0.06 (0.00, 1.73) | Inf (0.00, Inf) | 1.00 | 0.75 (0.28, 2.02) | 0.57 | 0.99 |
| *Bifidobacterium* | 4.12 (0.00, 27.43) | 1.42 (0.71, 2.82) | 0.32 | 1.24 (0.84, 1.85) | 0.28 | 0.31 |
| *Faecalibacterium* | 8.89 (0.00, 31.58) | 1.11 (0.50, 2.49) | 0.80 | 1.35 (0.87, 2.09) | 0.17 | 0.60 |
| *Prevotella* | 2.81 (0.00, 48.70) | 0.37 (0.11, 1.29) | 0.12 | 1.20 (0.84, 1.74) | 0.32 | 0.14 |
| *Escherichia* | 0.06 (0.00, 4.86) | NA (NA, NA) | 0.11 | 1.34 (0.61, 2.95) | 0.47 | 0.49 |

Adjusted for baseline age (<65 years old, ≥65 years old), sex (male or female), stage at diagnosis (I-II, III), tumor site (colon or rectum), smoking status (current, former, never), recruitment center (Heidelberg University Hospital, Germany or Huntsman Cancer Institute, Salt Lake City)

*Abbreviations*: OR, Odds Ratios; CI, Confidence Intervals; HCI, Huntsman Cancer Institute; HD, Heidelberg

.

**Supplemental Table 4:** Associations of alpha-diversity with cachexia onset at 6 months post-surgery excluding patients with antibiotic use in the ColoCare Study, n= 84

|  | OR (95% CI) | P-value |
| --- | --- | --- |
| **Alpha diversity metrics** |  |  |
| Shannon index | 1.84 (0.97, 3.51) | 0.06 |
| Observed Species | 1.65 (0.91, 3.00) | 0.10 |
| Faith’s PD | 1.67 (0.92, 3.05) | 0.09 |

Alpha diversity metrics were standardized to a mean of 0 and standard deviation of 1. Standardized alpha-diversity Z-scores were estimated: Z = (X-μ)/σ, where Z = Z-score, X =observed alpha-diversity value for a specific sample or patient, μ = average alpha-diversity value across all samples or the mean of the entire patient distribution, and σ = standard deviation of the alpha-diversity values across all samples or the entire patient distribution.

Adjusted for baseline age (<65 years old, ≥65 years old), sex (male or female), body mass index (BMI) in kg/m^2^, stage at diagnosis (I, II, III), tumor site (colon or rectum), smoking status (current, former, never), recruitment center (Heidelberg University Hospital, Germany or Huntsman Cancer Institute, Salt Lake City), NSAID/ Aspirin use (no, yes)

*Abbreviations*: OR, Odds Ratios; CI, Confidence Intervals.

**Supplemental Table 5**. Associations^1^ of *a priori*-selected bacterial relative abundances^2^ with cachexia onset at 6 months post-surgery excluding patients with antibiotic use in the ColoCare Study, n=84

|  | Mean (min-max) | OR (95% CI) | P-values |
| --- | --- | --- | --- |
| ***A priori*-selected bacteria associated with CRC** |  |  |  |
| *Bacteroides* | 9.30 (0.00, 64.49) | 0.89 (0.52, 1.49) | 0.66 |
| Clostridiales (Order) | 73.16 (18.47, 96.49) | 1.59 (0.73, 3.50) | 0.24 |
| *Dialister* | 0.88 (0.00, 6.39) | 0.81 (0.48, 1.33) | 0.41 |
| *Fusobacterium* | 0.18 (0.00, 10.77) | 0.98 (0.59, 2.09) | 0.93 |
| *Parvimonas* | 0.14 (0.00, 6.68) | 0.90 (0.49, 1.57) | 0.70 |
| *Peptostreptococcus* | 0.35 (0.00, 17.43) | 1.08 (0.65, 1.77) | 0.77 |
| *Porphyromonas* | 0.10 (0.00, 3.36) | 0.52 (0.25, 1.05) | 0.07 |
| *Prevotella* | 2.81 (0.00, 48.70) | 1.17 (0.80, 1.77) | 0.43 |
| ***A priori*-selected bacteria associated with cachexia in non-CRC patients** |  |  |  |
| *Actinomyces* | 0.05 (0.00, 0.88) | 0.80 (0.54, 1.15) | 0.24 |
| *Lactobacillus* | 0.70 (0.00, 36.87) | 0.94 (0.62, 1.40) | 0.77 |
| *Parabacteroides* | 0.89 (0.00, 4.83) | 0.94 (0.52, 1.71) | 0.85 |
| *Bacteroides* | 9.30 (0.00, 64.49) | 0.87 (0.52, 1.42) | 0.59 |
| *Veillonella* | 0.18 (0.00, 5.66) | 0.72 (0.43, 1.15) | 0.18 |
| *Megamonas* | 0.17 (0.00, 22.30) | 0.51 (0.02, 1.54) | 0.41 |
| *Peptococcus* | 0.06 (0.00, 1.73) | 0.87 (0.30, 2.93) | 0.79 |
| *Bifidobacterium* | 4.12 (0.00, 27.43) | 1.20 (0.84, 1.75) | 0.33 |
| *Faecalibacterium* | 8.89 (0.00, 31.58) | 1.38 (0.93, 2.23) | 0.13 |
| *Prevotella* | 2.81 (0.00, 48.70) | 1.23 (0.86, 1.81) | 0.26 |
| *Escherichia* | 0.06 (0.00, 4.86) | 1.44 (0.70, 4.10) | 0.40 |

^1^Odds ratios and 95% confidence intervals were estimated using multivariable logistic regression adjusted for baseline age (<65 years old, ≥65 years old), sex (male or female), stage at diagnosis (I, II, III), tumor site (colon or rectum), smoking status (current, former, never), recruitment center (Heidelberg University Hospital, Germany or Huntsman Cancer Institute, Salt Lake City), NSAID/ Aspirin use (no, yes), C-reactive protein (CRP) levels, mg/L (low CRP, high CRP), dietary fiber intake, grams/day (low fiber, high fiber) and energy intake, kcal (Group 1, Group 2).

^2^Bacteria relative abundances were transformed with the centered-log ratio transformation method. Includes all genera identified. Bacteria present in ≥50% of samples at an average relative abundance of ≥0.01%

*Abbreviations*: PD, Faith’s Phylogenetic Diversity; OR, Odds Ratios; CI, Confidence Intervals.

**Supplemental Table 6:** Associations^1^ of exploratory bacterial relative abundances^2^ with cachexia onset at 6 months post-surgery excluding patients with antibiotic use in the ColoCare Study, n=84

|  | Mean (min-max) | OR (95% CI) | P-values^3^ |
| --- | --- | --- | --- |
| **Exploratory-selected bacteria** |  |  |  |
| *Akkermansia* | 1.63 (0.00, 28.03) | 0.81 (0.55, 1.17) | 0.28 |
| *Alistipes* | 1.11 (0.00, 11.54) | 1.00 (0.65, 1.53) | 0.99 |
| *Anaerostipes* | 0.32 (0.00, 5.53) | 1.15 (0.69, 1.97) | 0.60 |
| *Biloxi* | 0.17 (0.00, 1.34) | 0.87 (0.43, 1.74) | 0.69 |
| *Blautia* | 14.51 (1.17, 42.81) | 1.02 (0.50, 2.01) | 0.96 |
| *Butyricicoccus* | 0.30 (0.00, 1.61) | 1.53 (0.76, 3.38) | 0.26 |
| *Clos_Clostridium* | 1.84 (0.00, 37.33) | 1.27 (0.77, 2.17) | 0.36 |
| *Collinsella* | 2.57 (0.00, 22.83) | 1.15 (0.78, 1.71) | 0.49 |
| *Coprococcus* | 5.45 (0.28, 19.75) | 1.62 (0.81, 3.39) | 0.18 |
| *Dorea* | 3.54 (0.00, 14.70) | 1.23 (0.54, 2.79) | 0.61 |
| *Eggerthella* | 0.12 (0.00, 1.49) | 1.45 (0.90, 2.44) | 0.14 |
| *Erys_Clostridium* | 0.11 (0.00, 4.01) | 1.82 (1.01, 3.28) | 0.04 |
| *Eubacterium* | 0.56 (0.00, 4.80) | 1.15 (0.77, 1.75) | 0.50 |
| *Faecalibacterium* | 8.89 (0.00, 31.58) | 1.48 (0.96, 2.30) | 0.08 |
| *Gemmiger* | 4.72 (0.00, 24.84) | 1.19 (0.81, 1.81) | 0.39 |
| *Lach_Clostridium* | 0.61 (0.00, 10.67) | 0.98 (0.62, 1.52) | 0.92 |
| *Lach_Ruminococcus* | 0.46 (0.00, 3.04) | 0.85 (0.48, 1.51) | 0.58 |
| *Lachnospira* | 1.49 (0.00, 10.07) | 1.37 (0.77, 2.63) | 0.31 |
| *Oscillospira* | 4.33 (0.00, 33.15) | 1.08 (0.67, 1.80) | 0.77 |
| *Phascolarctobacterium* | 0.55 (0.00, 4.62) | 0.90 (0.42, 1.81) | 0.77 |
| *Roseburia* | 8.03 (0.00, 29.75) | 1.22 (0.82, 1.85) | 0.34 |
| *Rumi_Ruminococcus* | 8.77 (0.00, 25.88) | 1.27 (0.89, 1.86) | 0.19 |
| *Ruminococcus* | 3.72 (0.00, 52.56) | 0.77 (0.40, 1.41) | 0.40 |
| *Streptococcus* | 1.19 (0.00, 25.10) | 1.28 (0.81, 2.08) | 0.31 |
| *Sutterella* | 0.59 (0.00, 3.20) | 0.79 (0.40, 1.52) | 0.48 |
| *Turicibacter* | 0.23 (0.00, 6.11) | 0.68 (0.36, 1.21) | 0.20 |

^1^Odds ratios and 95% confidence intervals were estimated using multivariable logistic regression adjusted for baseline age (<65 years old, ≥65 years old), sex (male or female), stage at diagnosis (I, II, III), tumor site (colon or rectum), smoking status (current, former, never), recruitment center (Heidelberg University Hospital, Germany or Huntsman Cancer Institute, Salt Lake City), NSAID/ Aspirin use (no, yes), C-reactive protein (CRP) levels, mg/L (low CRP, high CRP), dietary fiber intake, grams/day (low fiber, high fiber) and energy intake, kcal (Group 1, Group 2).

^2^Bacteria relative abundances were transformed with the centered-log ratio transformation method. Includes all genera identified. Bacteria present in ≥50% of samples at an average relative abundance of ≥0.01%

^3^P-values are corrected for multiple testing using the Bonferroni correction method at a significance level of 0.0019 (0.05/ 27).

*Abbreviations*: OR: Odds Ratios, CI: Confidence intervals; *Clos_Clostridium: Clostridiaceae (family) Clostridium; Erys_Clostridium: Erysipelotrichaceae (family) Clostridium; Lach_Clostridium: Lachnospiracea (family) Clostridium, Lach_Ruminococcus: Lachnospiracea (family) Ruminococcus; Rumi_Ruminococcus: Ruminococcaceae (family) Ruminococcus*

**Supplemental Table 7:** Associations of alpha-diversity with cachexia onset at 6 months post-surgery excluding patients who received neo-adjuvant treatment in the ColoCare Study, n= 76

|  | OR (95% CI) | P-value |
| --- | --- | --- |
| **Alpha diversity metrics** |  |  |
| Shannon index | 3.36 (1.13, 10.00) | **0.03** |
| Observed Species | 1.82 (0.84, 3.95) | 0.13 |
| Faith’s PD | 2.05 (0.91, 4.63) | 0.08 |

Alpha diversity metrics were standardized to a mean of 0 and standard deviation of 1. Standardized alpha-diversity Z-scores were estimated: Z = (X-μ)/σ, where Z = Z-score, X =observed alpha-diversity value for a specific sample or patient, μ = average alpha-diversity value across all samples or the mean of the entire patient distribution, and σ = standard deviation of the alpha-diversity values across all samples or the entire patient distribution.

Adjusted for baseline age (<65 years old, ≥65 years old), sex (male or female), body mass index (BMI) in kg/m^2^, stage at diagnosis (I, II, III), tumor site (colon or rectum), smoking status (current, former, never), recruitment center (Heidelberg University Hospital, Germany or Huntsman Cancer Institute, Salt Lake City), NSAID/ Aspirin use (no, yes)

*Abbreviations*: OR, Odds Ratios; CI, Confidence Intervals.

**Supplemental Table 8**. Associations^1^ of *a priori*-selected bacterial relative abundances^2^ with cachexia onset at 6 months post-surgery excluding patients who received neo-adjuvant treatment in the ColoCare Study, n= 76

|  | Mean (min-max) | OR (95% CI) | P-values |
| --- | --- | --- | --- |
| ***A priori*-selected bacteria associated with CRC** |  |  |  |
| *Bacteroides* | 9.30 (0.00, 64.49) | 0.78 (0.44, 1.41) | 0.41 |
| Clostridiales (Order) | 73.16 (18.47, 96.49) | 1.01 (0.41, 2.48) | 0.98 |
| *Dialister* | 0.88 (0.00, 6.39) | 0.87 (0.49, 1.55) | 0.64 |
| *Fusobacterium* | 0.18 (0.00, 10.77) | 0.91 (0.51, 1.64) | 0.75 |
| *Parvimonas* | 0.14 (0.00, 6.68) | 1.12 (0.55, 2.28) | 0.75 |
| *Peptostreptococcus* | 0.35 (0.00, 17.43) | 1.08 (0.63, 1.85) | 0.79 |
| *Porphyromonas* | 0.10 (0.00, 3.36) | 1.01 (0.32, 3.26) | 0.98 |
| *Prevotella* | 2.81 (0.00, 48.70) | 1.58 (0.89, 2.79) | 0.12 |
| ***A priori*-selected bacteria associated with cachexia in non-CRC patients** |  |  |  |
| *Actinomyces* | 0.05 (0.00, 0.88) | 0.85 (0.51, 1.43) | 0.55 |
| *Lactobacillus* | 0.70 (0.00, 36.87) | 0.95 (0.59, 1.54) | 0.84 |
| *Parabacteroides* | 0.89 (0.00, 4.83) | 0.82 (0.39, 1.70) | 0.59 |
| *Bacteroides* | 9.30 (0.00, 64.49) | 0.78 (0.44, 1.41) | 0.41 |
| *Veillonella* | 0.18 (0.00, 5.66) | 0.64 (0.35, 1.17) | 0.15 |
| *Megamonas* | 0.17 (0.00, 22.30) | 0.02 (0.00, Inf) | 1.00 |
| *Peptococcus* | 0.06 (0.00, 1.73) | 0.49 (0.14, 1.80) | 0.29 |
| *Bifidobacterium* | 4.12 (0.00, 27.43) | 1.13 (0.71, 1.80) | 0.60 |
| *Faecalibacterium* | 8.89 (0.00, 31.58) | 1.29 (0.79, 2.11) | 0.31 |
| *Prevotella* | 2.81 (0.00, 48.70) | 1.58 (0.89, 2.79) | 0.12 |
| *Escherichia* | 0.06 (0.00, 4.86) | 1.17 (0.51, 2.67) | 0.71 |

^1^Odds ratios and 95% confidence intervals were estimated using multivariable logistic regression adjusted for baseline age (<65 years old, ≥65 years old), sex (male or female), stage at diagnosis (I, II, III), tumor site (colon or rectum), smoking status (current, former, never), recruitment center (Heidelberg University Hospital, Germany or Huntsman Cancer Institute, Salt Lake City), NSAID/ Aspirin use (no, yes), C-reactive protein (CRP) levels, mg/L (low CRP, high CRP), dietary fiber intake, grams/day (low fiber, high fiber) and energy intake, kcal (Group 1, Group 2).

^2^Bacteria relative abundances were transformed with the centered-log ratio transformation method. Includes all genera identified. Bacteria present in ≥50% of samples at an average relative abundance of ≥0.01%

*Abbreviations*: PD, Faith’s Phylogenetic Diversity; OR, Odds Ratios; CI, Confidence Intervals.

**Supplemental Table 9:** Associations^1^ of exploratory bacterial relative abundances^2^ with cachexia onset at 6 months post-surgery excluding patients who received neo-adjuvant treatment in the ColoCare Study, n= 76

|  | Mean (min-max) | OR (95% CI) | P-values^3^ |
| --- | --- | --- | --- |
| **Exploratory-selected bacteria** |  |  |  |
| *Akkermansia* | 1.63 (0.00, 28.03) | 0.72 (0.44, 1.18) | 0.19 |
| *Alistipes* | 1.11 (0.00, 11.54) | 1.06 (0.68, 1.66) | 0.79 |
| *Anaerostipes* | 0.32 (0.00, 5.53) | 1.18 (0.60, 2.30) | 0.64 |
| *Biloxi* | 0.17 (0.00, 1.34) | 0.81 (0.39, 1.67) | 0.57 |
| *Blautia* | 14.51 (1.17, 42.81) | 0.72 (0.33, 1.58) | 0.41 |
| *Butyricicoccus* | 0.30 (0.00, 1.61) | 1.11 (0.52, 2.35) | 0.79 |
| *Clos_Clostridium* | 1.84 (0.00, 37.33) | 1.24 (0.73, 2.09) | 0.43 |
| *Collinsella* | 2.57 (0.00, 22.83) | 1.28 (0.77, 2.11) | 0.34 |
| *Coprococcus* | 5.45 (0.28, 19.75) | 1.16 (0.52, 2.61) | 0.72 |
| *Dorea* | 3.54 (0.00, 14.70) | 1.38 (0.57, 3.36) | 0.48 |
| *Eggerthella* | 0.12 (0.00, 1.49) | 1.57 (0.85, 2.92) | 0.15 |
| *Erys_Clostridium* | 0.11 (0.00, 4.01) | 2.42 (1.10, 5.33) | 0.03 |
| *Eubacterium* | 0.56 (0.00, 4.80) | 1.11 (0.72, 1.72) | 0.64 |
| *Faecalibacterium* | 8.89 (0.00, 31.58) | 1.29 (0.79, 2.11) | 0.31 |
| *Gemmiger* | 4.72 (0.00, 24.84) | 1.29 (0.81, 2.05) | 0.28 |
| *Lach_Clostridium* | 0.61 (0.00, 10.67) | 0.68 (0.38, 1.20) | 0.18 |
| *Lach_Ruminococcus* | 0.46 (0.00, 3.04) | 0.75 (0.39, 1.44) | 0.39 |
| *Lachnospira* | 1.49 (0.00, 10.07) | 1.28 (0.73, 2.24) | 0.39 |
| *Oscillospira* | 4.33 (0.00, 33.15) | 1.05 (0.59, 1.86) | 0.87 |
| *Phascolarctobacterium* | 0.55 (0.00, 4.62) | 1.08 (0.49, 2.37) | 0.85 |
| *Roseburia* | 8.03 (0.00, 29.75) | 1.23 (0.76, 1.99) | 0.39 |
| *Rumi_Ruminococcus* | 8.77 (0.00, 25.88) | 1.22 (0.79, 1.88) | 0.37 |
| *Ruminococcus* | 3.72 (0.00, 52.56) | 0.85 (0.39, 1.83) | 0.67 |
| *Streptococcus* | 1.19 (0.00, 25.10) | 1.23 (0.72, 2.09) | 0.45 |
| *Sutterella* | 0.59 (0.00, 3.20) | 0.87 (0.47, 1.62) | 0.66 |
| *Turicibacter* | 0.23 (0.00, 6.11) | 0.53 (0.25, 1.13) | 0.10 |

^1^Odds ratios and 95% confidence intervals were estimated using multivariable logistic regression adjusted for baseline age (<65 years old, ≥65 years old), sex (male or female), stage at diagnosis (I, II, III), tumor site (colon or rectum), smoking status (current, former, never), recruitment center (Heidelberg University Hospital, Germany or Huntsman Cancer Institute, Salt Lake City), NSAID/ Aspirin use (no, yes), C-reactive protein (CRP) levels, mg/L (low CRP, high CRP), dietary fiber intake, grams/day (low fiber, high fiber) and energy intake, kcal (Group 1, Group 2).

^2^Bacteria relative abundances were transformed with the centered-log ratio transformation method. Includes all genera identified. Bacteria present in ≥50% of samples at an average relative abundance of ≥0.01%

^3^P-values are corrected for multiple testing using the Bonferroni correction method at a significance level of 0.0019 (0.05/ 27).

*Abbreviations*: OR: Odds Ratios, CI: Confidence intervals; *Clos_Clostridium: Clostridiaceae (family) Clostridium; Erys_Clostridium: Erysipelotrichaceae (family) Clostridium; Lach_Clostridium: Lachnospiracea (family) Clostridium, Lach_Ruminococcus: Lachnospiracea (family) Ruminococcus; Rumi_Ruminococcus: Ruminococcaceae (family) Ruminococcus*

**Supplemental Table 10:** Associations^1^ of exploratory bacterial presence/absence^2^ with cachexia onset at 6 months post-surgery in the ColoCare Study, n=103

|  | OR (95% CI) | P-values |
| --- | --- | --- |
| **Exploratory-selected bacteria** |  |  |
| *Fusobacterium* | 0.16 (0.05, 0.54) | 0.003 |
| *Eubacterium* | 0.17 (0.04, 0.67) | 0.01 |
| *Lactococcus* | 3.63 (1.14, 11.5) | 0.03 |
| *Akkermansia* | 3.59 (1.17, 11.02) | 0.03 |
| *Clostridium* | 0.33 (0.12, 0.93) | 0.04 |
| *Defluvitalea* | 8.56 (1.00, 73.37) | 0.05 |
| *Parvimonas* | 0.34 (0.11, 1.00) | 0.05 |

^1^Odds ratios and 95% confidence intervals were estimated using multivariable logistic regression adjusted for baseline age (<65 years old, ≥65 years old), sex (male or female), stage at diagnosis (I, II, III), tumor site (colon or rectum), smoking status (current, former, never), recruitment center (Heidelberg University Hospital, Germany or Huntsman Cancer Institute, Salt Lake City), NSAID/ Aspirin use (no, yes), C-reactive protein (CRP) levels, mg/L (low CRP, high CRP), dietary fiber intake, grams/day (low fiber, high fiber) and energy intake, kcal (Group 1, Group 2).

^2^Presence was defined as a genus being detected (i.e., non-zero relative abundance) in a given sample. Prevalence calculated as the proportion of samples in which each genus was detected. Genera with a prevalence between 5% to 95% across all samples were included in the analysis

*Abbreviations*: OR: Odds Ratios, CI: Confidence intervals

**Supplemental Table 11**: Alpha-diversity distribution in the study population, n= 103

| Characteristics | Summary measure | Cachectic, n=44 | Non-cachectic | p-value^1^ |
| --- | --- | --- | --- | --- |
| Shannon | Median (IQR) | 4.09 (3.80, 4.27) | 3.90 (3.45, 4.12) | 0.01 |
|  | Mean ± SD | 4.05 ± 0.32 | 3.78 ± 0.52 |  |
| Observed | Median (IQR) | 198 (158, 226) | 170 (140, 195) | 0.02 |
|  | Mean ± SD | 197 ± 52.30 | 167 ± 53.30 |  |
| Faith’s PD | Median (IQR) | 643 (529, 721) | 584 (503, 644) | 0.03 |
|  | Mean ± SD | 640 ± 1.24 | 566 ± 1.40 |  |

^1^Wilcoxon rank sum test
