# Supplementary material for: Pre-surgery gut microbial diversity and abundance are associated with post-surgery onset of cachexia in colorectal cancer patients: the ColoCare Study
Source: Cancer Causes Control. 2025 Sep 4;36(12):1795–812. doi: 10.1007/s10552-025-02042-y (PMC12478455; doi:10.1007/s10552-025-02042-y)
Supplement: Supplementary file 2 — Supplementary file2 (DOCX 295 kb) [file 10552_2025_2042_MOESM2_ESM.docx]

**
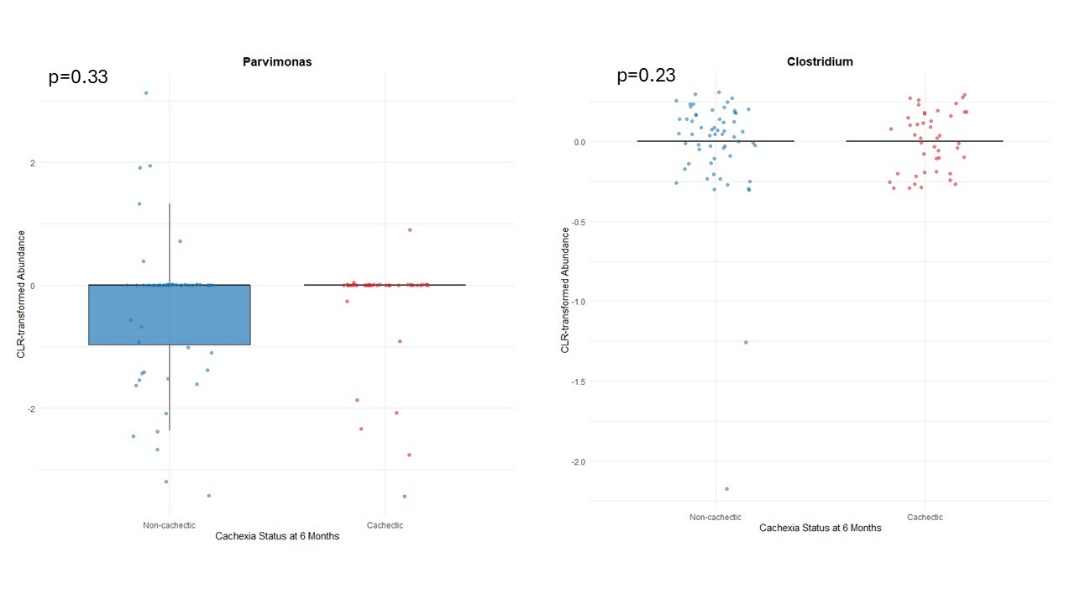

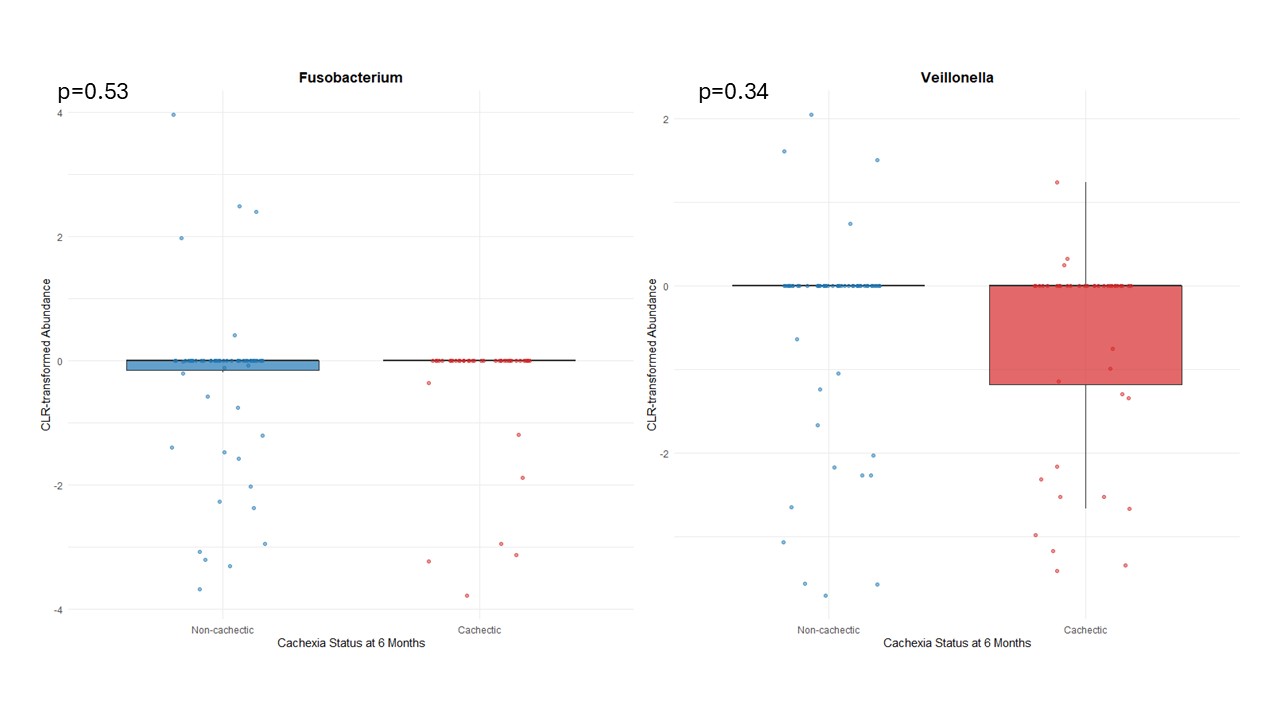

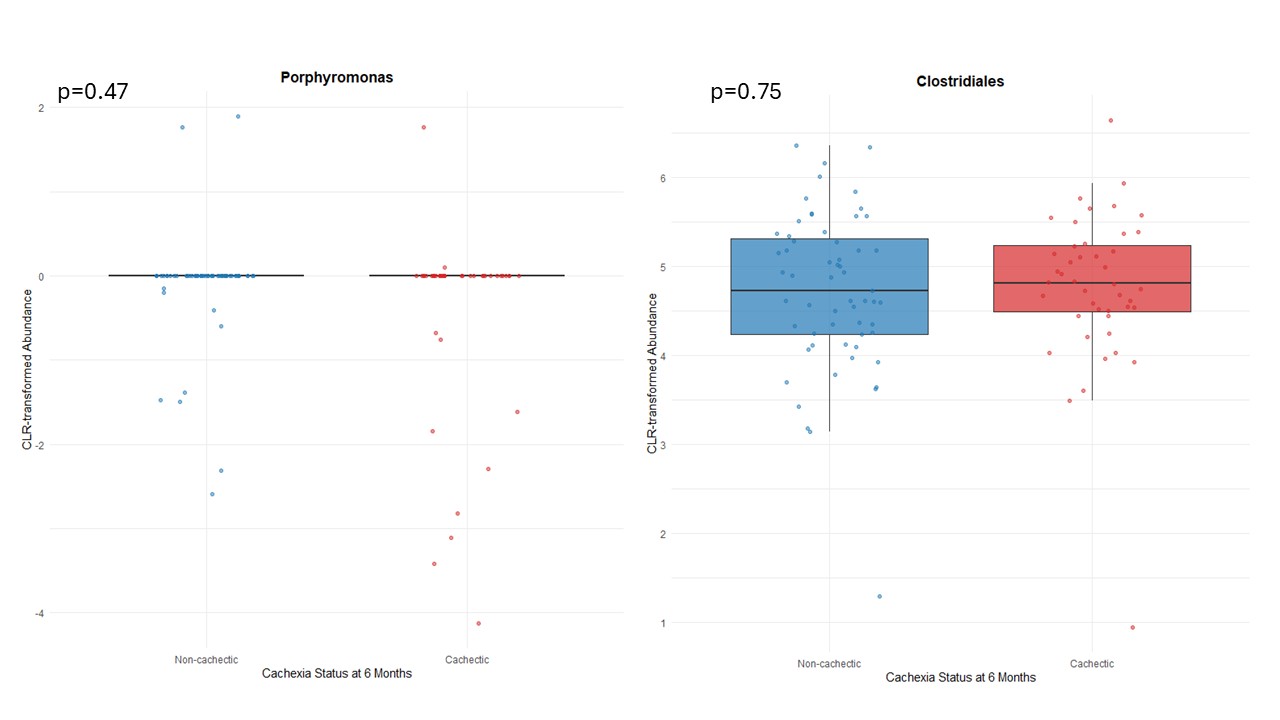

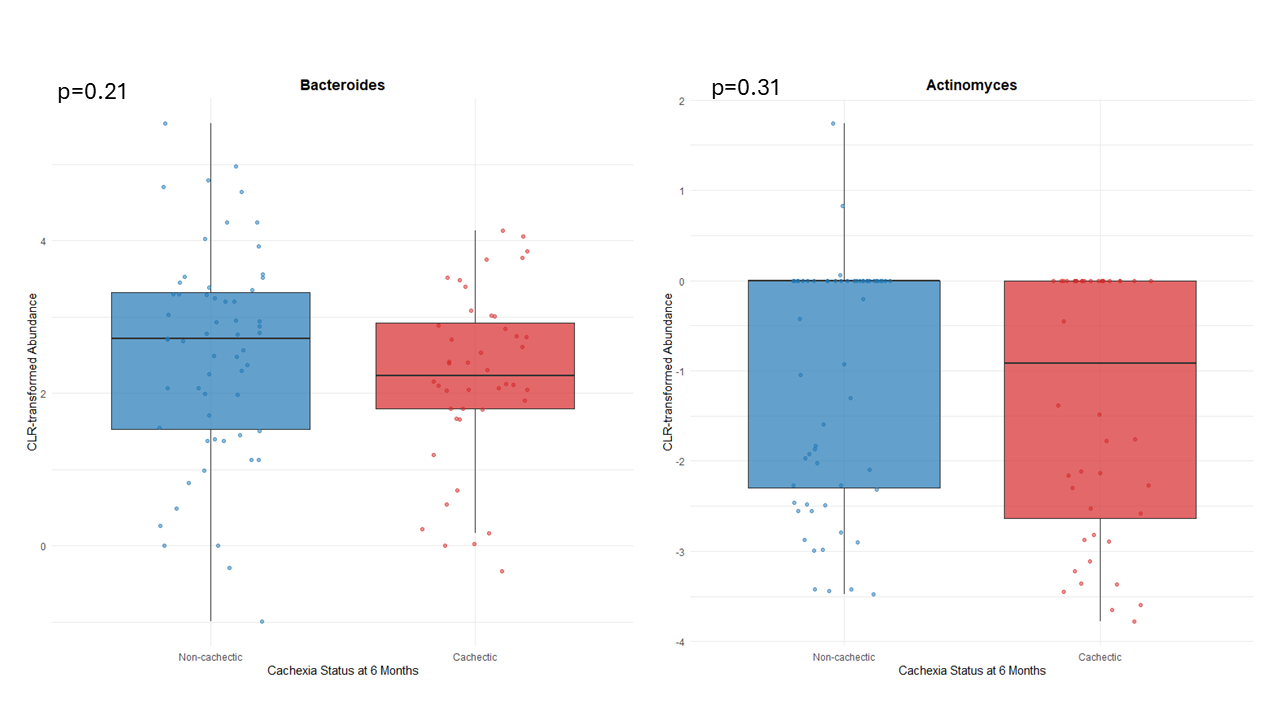
Supplemental Figure 1.** Comparison of genus-level relative abundances of a priori selected bacteria between cachectic and non-cachectic patients.

**
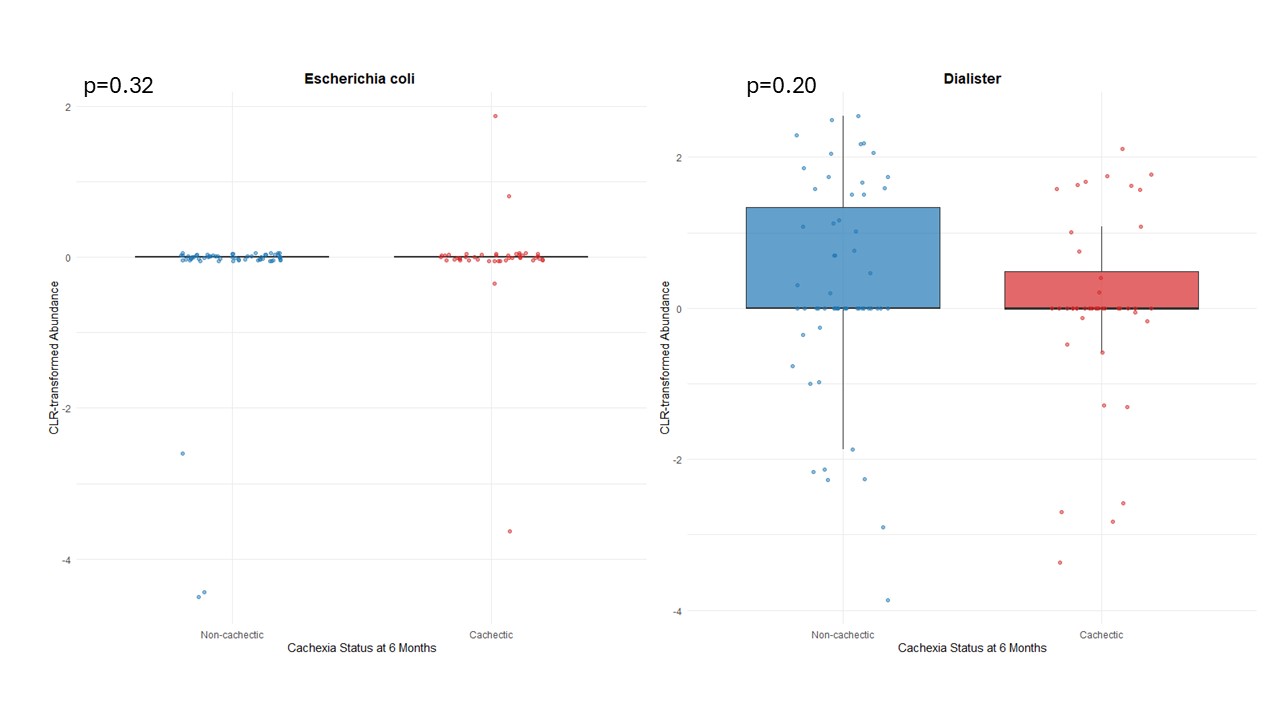
**

Wilcoxon rank sum test was used to compare genus-level relative abundances of a priori selected bacteria between cachectic and non-cachectic patients
